# Development and Evaluation of a 9K SNP Array for Peach by Internationally Coordinated SNP Detection and Validation in Breeding Germplasm

**DOI:** 10.1371/journal.pone.0035668

**Published:** 2012-04-20

**Authors:** Ignazio Verde, Nahla Bassil, Simone Scalabrin, Barbara Gilmore, Cynthia T. Lawley, Ksenija Gasic, Diego Micheletti, Umesh R. Rosyara, Federica Cattonaro, Elisa Vendramin, Dorrie Main, Valeria Aramini, Andrea L. Blas, Todd C. Mockler, Douglas W. Bryant, Larry Wilhelm, Michela Troggio, Bryon Sosinski, Maria José Aranzana, Pere Arús, Amy Iezzoni, Michele Morgante, Cameron Peace

**Affiliations:** 1 Fruit Tree Research Center, Rome, Italy; 2 United States Department of Agriculture-Agricultural Research Service, National Clonal Germplasm Repository, Corvallis, Oregon, United States of America; 3 Istituto di Genomica Applicata, Udine, Italy; 4 Illumina Inc., Hayward, California, United States of America; 5 School of Agricultural, Forest and Environmental Sciences, Clemson University, Clemson, South Carolina, United States of America; 6 IRTA, Centre de Recerca en Agrigenòmica CSIC-IRTA-UAB-UB, Campus UAB, Cerdanyola del Vallès (Bellaterra), Barcelona, Spain; 7 Department of Horticulture, Michigan State University, East Lansing, Michigan, United States of America; 8 Department of Horticulture & Landscape Architecture, Washington State University, Pullman, Washington, United States of America; 9 The Donald Danforth Plant Science Center, St. Louis, Missouri, United States of America; 10 Department of Botany and Plant Pathology and Center for Genome Research and Biocomputing, Oregon State University, Corvallis, Oregon, United States of America; 11 Intuitive Genomics, Inc., St. Louis, Missouri, United States of America; 12 Oregon Health Sciences University, Portland, Oregon, United States of America; 13 The Istituto Agrario di San Michele all'Adige Research and Innovation Centre, Foundation Edmund Mach, San Michele all'Adige, Italy; 14 Department of Horticultural Science, North Carolina State University, Raleigh, North Carolina, United States of America; 15 Dipartimento di Scienze Agrarie e Ambientali, University of Udine, Udine, Italy; Pennsylvania State University, United States of America

## Abstract

Although a large number of single nucleotide polymorphism (SNP) markers covering the entire genome are needed to enable molecular breeding efforts such as genome wide association studies, fine mapping, genomic selection and marker-assisted selection in peach [*Prunus persica* (L.) Batsch] and related *Prunus* species, only a limited number of genetic markers, including simple sequence repeats (SSRs), have been available to date. To address this need, an international consortium (The International Peach SNP Consortium; IPSC) has pursued a coordinated effort to perform genome-scale SNP discovery in peach using next generation sequencing platforms to develop and characterize a high-throughput Illumina Infinium® SNP genotyping array platform. We performed whole genome re-sequencing of 56 peach breeding accessions using the Illumina and Roche/454 sequencing technologies. Polymorphism detection algorithms identified a total of 1,022,354 SNPs. Validation with the Illumina GoldenGate® assay was performed on a subset of the predicted SNPs, verifying ∼75% of genic (exonic and intronic) SNPs, whereas only about a third of intergenic SNPs were verified. Conservative filtering was applied to arrive at a set of 8,144 SNPs that were included on the IPSC peach SNP array v1, distributed over all eight peach chromosomes with an average spacing of 26.7 kb between SNPs. Use of this platform to screen a total of 709 accessions of peach in two separate evaluation panels identified a total of 6,869 (84.3%) polymorphic SNPs.

The almost 7,000 SNPs verified as polymorphic through extensive empirical evaluation represent an excellent source of markers for future studies in genetic relatedness, genetic mapping, and dissecting the genetic architecture of complex agricultural traits. The IPSC peach SNP array v1 is commercially available and we expect that it will be used worldwide for genetic studies in peach and related stone fruit and nut species.

## Introduction

Dissection of the genetic components underlying complex agricultural traits in plants has so far used mainly experimental bi-parental crosses and a limited number of genetic markers. The growing number of draft genome sequences of many species coupled with Next Generation Sequencing (NGS) technologies has quickly changed the paradigm of plant genome analysis. Alignment of the short NGS reads obtained from diverse and unrelated varieties to a reference genome allows the identification of large numbers of Single Nucleotide Polymorphisms (SNPs) and small Deletion/Insertion Polymorphisms (DIPs) and the contextual estimation of their minor allele frequencies (MAF).

Simultaneous genotyping of hundreds of thousands of SNPs in a single assay has become feasible due to innovative combinations of assay and array platform multiplexing [1]. Illumina's Infinium® BeadArray Technology platform is an extremely high-throughput SNP genotyping system that allows the detection of up to 2.5 million SNPs per single DNA sample [2]. Multiplex SNP genotyping enables cost effective marker-assisted selection strategies, whole genome fingerprinting, genome-wide association studies (GWAS), map-based gene cloning and population-based analyses. The availability of such tools fosters the application of GWAS in plants and animals [3], [4], [5], [6] and provides the opportunity to apply genomic selection (GS) methods to agricultural species, including *Prunus*. High-density SNP genotyping arrays have been designed for several domestic animals including cattle [3], pig [4] and chicken [5]; arrays are being developed in several plant species including apple [6], maize, tomato, and cherry (http://www.illumina.com/agriculture).

In peach [*Prunus persica* L. (Batsch)] and related *Prunus* species, QTL studies have been conducted using experimental bi-parental crosses and a limited number of genetic markers [7], [8], [9], [10], [11], [12], [13]. A recent association study by Aranzana et al. [14] using a limited number (50) of SSR markers in unrelated accessions from American and European origin indicated that linkage disequilibrium (LD) in peach is quite high, up to 13–15 cM with stratification of peach accessions into at least three sub-populations. This study suggests that a small number of markers (about 600) might be sufficient to scan the peach genome. However, the peach gene pool used in this study [14] is known to have a narrow genetic base [15] in comparison with Eastern germplasm [16] where LD is likely to have a lower level of conservation. Moreover, studies in grape [17], [18] and maize [19] suggest that SNPs estimate a much lower decay of LD than SSRs. Hence, we suggest that a higher number of SNP markers covering the entire genome would be necessary to scan the peach genome and to perform GWAS.

The availability of the peach reference genome sequence recently released by the International Peach Genome Initiative [20] facilitated genome wide variant detection and the development of dedicated genomic tools. This and the ability to acquire massive sequence datasets from next generation sequencers, allowed the efficient identification of a large number of genetic markers, such as SNPs and small DIPs, enabling the development of a SNP array in this important horticultural crop. We describe here how members of The International Peach SNP Consortium (IPSC), that includes institutions from the U.S., Italy, and Spain, have worked together to identify genome-wide sequence variation and to develop a moderate-density peach high-throughput Infinium® SNP genotyping platform relevant for worldwide peach breeding germplasm, utilizing SNPs discovered using next generation sequencing platforms.

## Materials and Methods

### Whole genome re-sequencing of peach breeding accessions

A SNP detection panel of 56 peach breeding-relevant accessions assembled with the goal of achieving an efficient coverage of the genetic background of cultivated peach (Table 1) was used for low-depth genome re-sequencing. The accessions were founders, intermediate ancestors, and important breeding parents used in international peach breeding programs, chosen both for the significance of their contribution to breeding germplasm according to pedigree records, and for genetic diversity based upon relatedness estimates from SSR studies [21], [14], (Verde I. unpublished data). Accessions were divided into 12 pools. For each accession in pools 1 through 11, paired-end libraries were prepared as recommended by Illumina (Illumina Inc., San Diego, CA, USA) separately at the USDA-ARS-National Clonal Germplasm Repository (NCGR) and the Istituto di Genomica Applicata (IGA, Udine, Italy) laboratories. In summary, library preparations were performed using minor modifications of the Illumina DNA-seq Sample Preparation protocol (Illumina, Inc., San Diego, CA). Briefly, 1–3 µg of genomic DNA was sheared by sonication using Diagenode's Biorupter XL sonicator system (Sparta, NJ, USA). This was followed by standard blunt-ending and ‘A’ was performed. Then, Illumina adapters with indexes (3 bp or 6 bp, at the NCGR and IGA, respectively) were ligated to the ends of the fragments. After the ligation reaction and separation of un-ligated adapters, samples were amplified by PCR to selectively enrich for those fragments in the library with adapter molecules at both ends. The samples were quantitated and quality tested using the NanoDrop ND-1000 UV-Vis Spectrophotometer (Thermo Scientific, Wilmington, DE, USA) and Agilent 2100 Bioanalyzer (Agilent Technologies, Santa Clara, CA). Libraries were pooled in equimolar ratios to yield a total concentration of 10 nM. Aliquots of pooled libraries (5 pmol) were processed with the Illumina Cluster Generation Station, following the manufacturer's recommendations. Pools were sequenced in one lane of Illumina GA II with 94 cycles per read at the Istituto di Genomica Applicata (IGA, Udine, Italy) for pools 1–5 and with 80 cycles per read at the Center for Genome Research and Biocomputing (CGRB, Oregon State University, Corvallis, OR, USA) for pools 6–11, as specified in Table 1. Libraries for accessions in pool 12 were constructed for 454 GS FLX sequencing with MID-labeled libraries. Nuclear DNA from the eight accessions of pool 12 was digested with *Alu*1 and size-selected for 400–500 bp fragments. At IGA the CASAVA 1.7.0 version of the Illumina pipeline was used and at the OSU CGRB the CASAVA 1.6.0 version of the Illumina pipeline was used. Raw sequences were retrieved and kept separate for each accession and then aligned to the Peach v1.0 reference genome [20] using CLC Genomics Workbench (CLC Bio, Aarhus, Denmark) at IGA and *Soap2* [22] at the CGRB. In this paper, “chromosome" refers to one of the eight pseudomolecules (scaffolds) of the Peach v1.0 reference genome.

**Table 1:** Accessions of peach, almond and peach x almond hybrid sequenced at the Istituto di Genomica Applicata (IGA, Udine, Italy) (pools 1–5), the Center for Genome Research and Biocomputing (CGRB, Oregon State University, Corvallis, OR, USA) (pools 6–11), and IRTA (Centre de Recerca en Agrigenòmica CSIC-IRTA-UAB, Spain) (pool 12).

Pool	Accession	Adaptors	Read length (bp)	Read count (million)	Coverage of peach genome
1	‘Armking’	CACAGT	94	5.85	2.42
1	‘Big Top’	CGAGAT	94	3.55	1.47
1	‘Fidelia’	ATGGCT	94	6.47	2.68
1	‘Flordastar’	GCATAG	94	7.27	3.01
1	‘Silver Rome’	CATTCG	94	8.35	3.45
1	‘Weinberger’	ACACTG	94	9.72	4.02
2	‘Babygold 8’	TTGCGA	93	5.60	2.29
2	‘Elberta’	CAGTAC	93	5.63	2.30
2	‘Maruja’	TGCAAC	93	8.52	3.49
2	‘Maycrest’	ACTAGC	93	8.61	3.52
2	‘Oro A’	GAGCAA	93	7.20	2.95
2	‘Stark Red Gold’	GCTACA	93	6.37	2.61
3	‘Circe’	CATTCG	93	9.23	3.78
3	‘Imera’	GCATAG	93	5.92	2.42
3	‘Percoca di Romagna 7’	ATGGCT	93	4.27	1.75
3	‘Pillar’	ACACTG	93	1.40	0.57
3	‘S 2678’	CGAGAT	93	10.15	4.15
3	‘Stark Saturn’	CACAGT	93	7.45	3.05
4	‘Kamarat’	ACTAGC	93	9.63	3.94
4	‘Leonforte 1’	GAGCAA	93	2.32	0.95
4	‘Sahua Hong Pantao’	GCTACA	93	19.20	7.86
4	‘Shen Zhou Mitao’	TTGCGA	93	12.54	5.13
4	‘Tabacchiera’	TGCAAC	93	0.56	0.23
4	‘Tudia’	CAGTAC	93	7.43	3.04
5	‘GF677’^1^	CGAGAT	93	9.22	3.77
5	‘Kurakata Wase’	ATGGCT	93	6.75	2.76
5	‘Quetta’	CACAGT	93	12.76	5.22
5	‘S6699’	ACACTG	93	4.90	2.01
6	‘Admiral Dewey’	GGGT	80	2.42	0.85
6	‘Babcock’	CCAT	80	3.19	1.12
6	‘Elberta’	AGCT	80	0.64	0.23
6	‘Slappey’	TCCT	80	2.02	0.71
7	‘Bolinha’	AGCT	80	3.55	1.25
7	‘Carmen’	GGGT	80	1.66	0.58
7	‘Chinese Cling’	TCCT	80	2.50	0.88
7	‘Mayflower’	CCAT	80	1.35	0.47
8	‘Diamante’	AGCT	80	2.11	0.74
8	‘J.H. Hale’	TCCT	80	3.18	1.12
8	‘Rio Oso Gem’	CCAT	80	2.57	0.91
8	‘Yellow St. John’	GGGT	80	1.35	0.48
9	‘Dixon’	GGGT	80	1.25	0.44
9	‘Early Crawford’	TCCT	80	3.89	1.37
9	‘Florida Prince’	CCAT	80	1.85	0.65
9	‘Nonpareil’^2^	AGCT	80	2.52	0.89
10	‘Dr. Davis’	GGGT	80	2.31	0.81
10	‘Nemaguard’	AGCT	80	2.38	0.84
10	‘O'Henry’	TCCT	80	4.28	1.51
10	‘Okinawa’	CCAT	80	2.15	0.76
11	‘Georgia Belle’	AACT	80	14.42	5.08
11	‘Lovell’	GTGT	80	6.55	2.30
11	‘Lovell’	CCTT	80	0.03	0.01
11	‘Oldmixon Free’	TGGT	80	3.26	1.15
12	‘Big Top’	ACACGTAGTAT	330	0.20	0.29
12	‘Binaced’	ACACGACGACT	355	0.16	0.26
12	‘Catherina’	ACACTACTCGT	288	0.17	0.22
12	‘Elegant Lady’	ACGACACGTAT	243	0.19	0.20
12	‘Nectaross’	ACGAGTAGACT	275	0.19	0.23
12	‘O'Henry’	ACGCGTCTAGT	289	0.15	0.18
12	‘Sweet Cap’	ACGTACACACT	251	0.16	0.18
12	‘Venus’	ACGTACTGTGT	278	0.15	0.19

1 Peach x almond hybrid;

2 Almond accession.

Pools 1–11 were sequenced with the Illumina Genome Analyzer while pool 12 was sequenced with the Roche 454 platform. Adaptors were used for retrieving accession-specific sequences from pools.

### SNP detection

**Figure 1:**
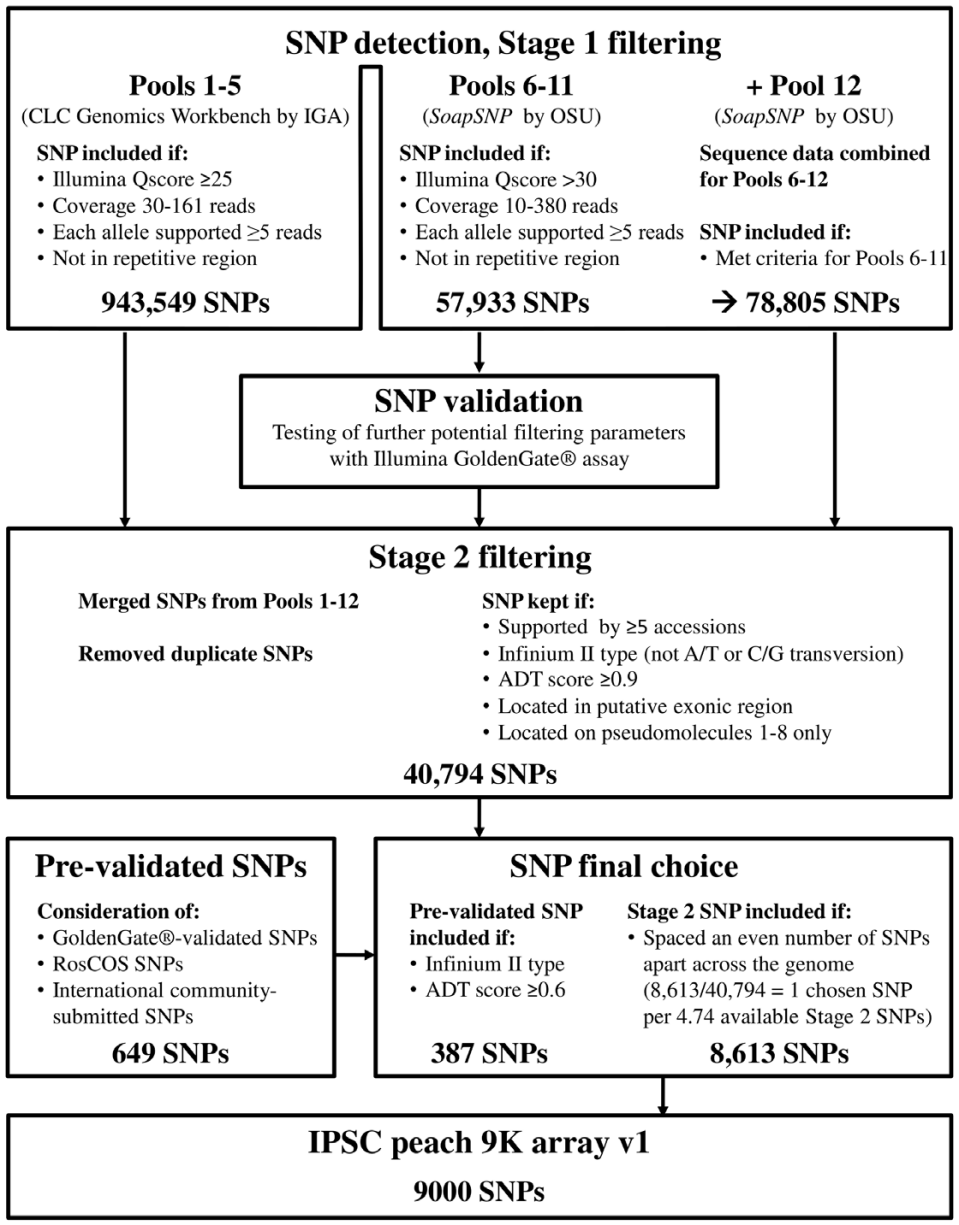
Workflow for SNP detection, validation, filtering, and final choice employed for development of the International Peach SNP Consortium (IPSC) peach 9 K SNP array v1.

SNP detection and filtering followed a multi-step procedure (Figure 1). SNPs from sequences generated at IGA (pools 1–5) were detected using CLC Genomics Workbench (CLC Bio, Aarhus, Denmark), using default parameters for filtering except: (1) minimum Illumina quality score (Qscore) was 25; (2) minimum coverage of 30 reads and maximum coverage was 2.5× average coverage, corresponding to 161 reads; and (3) a minimum of five reads supported the presence of the minor allele in the accessions. SNPs in repetitive regions were also removed with internal scripts. SNPs from the 23 CGRB-sequenced accessions (pools 6–11) and from the eight accessions in pool 12 were detected using *SoapSNP* (http://soap.genomics.org.cn/soapsnp.html) essentially as recommended by Li [22]. In filtering for pools 6–12, SNPs were kept if: (1) the Illumina Qscore was more than 30 (except for pool 12 for which an Illumina Qscore could not be obtained); (2) the maximum number of reads for either allele across all accessions was less than the average read depth of all SNPs plus three standard deviations, 380 in this case; (3) a minimum of five reads supported the presence of the minor allele in the accessions, providing a minimum coverage of 10 reads for the SNP; and (4) the average copy number of the SNP flanking region was less than two, corresponding to non-repetitive regions of the genome. These detection and filtering efforts yielded “Stage 1 SNPs" (Figure 1).

We compared the SNP calls in three of four pairs of accessions independently sequenced in different labs (Table 1). SNP calls were not compared for ‘Lovell’ because of low amount of sequence generated by one sample (0.01× coverage). For the remaining three cultivars, ‘Elberta’ (2.3× Illumina by IGA vs. 0.23× Illumina by CGRB), ‘Big Top’ (1.47× Illumina by IGA vs. 0.29× 454 by IRTA), and ‘O'Henry’ (1.51× Illumina by CGRB vs. 0.18× 454 by IRTA) (Table 1) the SNP calls were compared when they belonged to the 320,747 SNPs supported by >4 accessions in Stage 2 filtering (Figure 1).

### SNP validation with GoldenGate® assay

A set of 96 SNPs was chosen from the Stage 1 SNPs from pools 6–11 to validate the efficiency of SNP detection and adjust subsequent filtering parameters (Figure S1). The initial selection comprised 74 Stage 1 SNPs evenly spread over the eight pseudomolecules representing the haploid chromosomes and linkage groups (LGs) of the Peach v1.0 ‘dhLovell’ genome assembly [20]. One SNP was chosen to be located within 200 kb of each end of each LG. SNPs chosen between these ends were then evenly spaced along each LG according to their total genetic distance [23], corresponding to one SNP every 2–5 Mb. The spacing across all LGs averaged 3.28 Mb (standard deviation of 0.67 Mb), with a minimum average of 2.51 Mb (±0.01 Mb) for LG7 and a maximum average of 4.44 Mb (±0.01 Mb) for LG2. Approximately 40% of the 74 chosen SNPs were in exons (CDS) of annotated genes, 20% in introns, 20% in 5′ or 3′ untranslated regions (UTR, outside genes but within 2 kb of start or stop codons), and the final 20% in intergenic regions. Fourteen more SNPs spanned a 774 kb region near the end of LG4, at a major trait locus associated with fruit texture (*Freestone-Melting flesh* [*F-M*] locus) [24], at an average spacing of 60 kb (ranging from 8 kb to 113 kb). The final eight validation SNPs targeted candidate genes for the *Y* locus on LG1 (four SNPs at 123, 270, and 341 kb intervals), the *Cs* locus on LG3 (two SNPs 38 kb apart), and the *G* locus on LG5 (two SNPs 272 kb apart) according to the genomic positioning reported by Dirlewanger [23]. While trait locus-targeted SNPs were chosen for variation in genic regions when possible, preference was given to achieving uniform target spacing in designated windows. Approximately 20% of the 96 SNPs chosen were planned to be accession-specific, i.e., their minor allele would be detected in only one re-sequenced accession of the detection panel (for which data available at the time included only accessions from pools 6–11). Sixteen of the evenly spaced SNPs and four of the trait locus-targeted SNPs met this criterion. Accession-specific SNPs were from nine peach accessions, with the almond ‘Nonpareil’ and the peach reference cultivar ‘dhLovell’ having five and six such SNPs, respectively. The 96 SNPs also deliberately included a wide range of MAFs.

To test the variables of genomic location, genic location, and MAF for their effects on genotyping efficiency, a validation panel of 160 *Prunus* accessions (54 peach cultivars, three interspecific hybrid cultivars, three almond cultivars, 59 breeding selections of peach and related species, and 41 seedlings of breeding populations as listed in Table S1) was screened with the 96-SNP subset, using the GoldenGate® assay. Individuals in the validation panel were founders, intermediate ancestors, important breeding parents, and seedlings of modern peach cultivars, forming a complex pedigree structure linking much of the world's cultivated peach crop and including the 23 accessions in pools 6–11 of the detection panel. The validation panel also included the T×E bin-mapping set of six ‘Texas’ almond×‘Earlygold’ peach seedlings and ‘Earlygold’ [25] to enable validation of SNP genomic locations. For a snapshot of SNP polymorphisms in breeding populations, five progenies of six F_1_ seedlings were included, with one progeny set each from U.S. peach breeding programs in Arkansas, South Carolina, and Texas, and two from California. Genomic DNA was isolated from each accession using the E-Z 96 Tissue DNA Kit (Omega Bio-Tek, Inc., Norcross, GA, USA). DNA was quantitated with the Quant-iT™ PicoGreen® Assay (Life Technologies, Grand Island, NY, USA), using the Victor multiplate reader (Perkin Elmer Inc., San Jose, CA, USA). Concentrations were adjusted to a minimum of 50 ng/µl, in 5 µl aliquots and were submitted to the Research Technology Support Facility at Michigan State University (East Lansing, MI, USA), where the GoldenGate® assay was performed following the manufacturer's protocol (Illumina Inc.). After amplification, PCR products were hybridized to VeraCode microbeads via the address sequence, for detection on a VeraCode BeadXpress Reader. SNP genotypes were scored with the Genotyping Module of GenomeStudio Data Analysis software (Illumina Inc.).

### SNP final choice

Stage 1 SNPs from pools 1–5 and pools 6–12 were independently converted to Illumina Assay Design Tool (ADT) format with custom scripts and scored by Illumina. “Infinium® I"-type SNPs (A/T or C/G transversions) were removed, as well as SNPs with any failure code or ADT score <0.9. Then the two datasets were merged, and duplicates were removed with custom scripts. The remaining SNPs were filtered by removing those: (1) where the last 25 bp of the 50 bp probe were duplicated; (2) supported by less than five accessions; (3) not in predicted coding regions; and (4) not located on one of the first eight pseudomolecules representing the eight peach chromosomes. This process yielded “Stage 2 SNPs" (Figure 1).

Pre-validated SNPs were obtained from several sources: (1) polymorphic SNPs from the GoldenGate® validation activity; (2) peach RosCOS SNPs [26]; and (3) SNPs requested for inclusion by the international peach genomics community. Pre-validated SNPs were filtered to remove Infinium® I SNP types and those with an ADT score <0.6.

For the final choice of 9,000 SNPs for the IPSC peach 9 K SNP array v1, filtered pre-validated SNPs were automatically included. For the remainder, SNPs were chosen to provide an even spacing across the genome, corresponding to one SNP selected for every 4.74 Stage 2 SNPs (40,794/8,613).

### SNP array evaluation

The IPSC peach 9 K SNP Infinium® II array v1 was evaluated using 709 accessions divided in two independent evaluation panels, one panel from the European Union (EU) and the other from the USA (US). The EU panel comprised 232 accessions, of which 229 were peach cultivars and three were wild related *Prunus* species or their hybrids with peach. The US panel comprised 479 accessions that included pedigree-linked cultivars, breeding lines, and seedlings (Table S2). Overall, selected material comprised cultivars (45%), advanced selections (4%) and seedlings (51%). Accessions with pure peach and almond ancestry accounted for 82% and 2%, respectively, while 16% of genotyped material had interspecific backgrounds with almond (7%), and peach and almond wild relatives, 5% and 4%, respectively, in their pedigrees. Some US panel accessions were related *Prunus* species or were known interspecific hybrids: 5% had peach-related (*P. davidiana* and *P. mira*) ancestry, 10% had almond (*P. dulcis*), and 3% had almond-related (*P. argentea* and *P. scoparia*) ancestry. Genomic DNA extraction and quantitation were conducted as described above for the SNP validation panel for the U.S. accessions. For the EU panel, genomic DNA was extracted using the DNeasy Plant Mini Kit (Qiagen) and quantitated using a Fluoroskan Ascent (Thermo Scientific, Finland) microplate reader. The IPSC array, employing exclusively Illumina Infinium® II design probes and dual color channel assays (Infinium HD Assay Ultra, Illumina), was used for genotyping, following the manufacturer's recommendations. SNP genotypes were scored with the Genotyping Module of the GenomeStudio Data Analysis software (Illumina, Inc.). A GenTrain score of >0.4 and a GenCall 10% of >0.2 were applied to remove most SNPs that did not cluster (homozygous), or had ambiguous clustering. SNPs that did not cluster for more than 50% of samples were also eliminated from further consideration. The threshold of allowed No Calls (failed genotyping) was ‘relaxed’ in anticipation of the presence of null alleles for some SNPs contributed by non-peach species.

## Results

### SNP detection

A total of 25.4 Gb of DNA sequence (111.7× coverage of the peach genome) was obtained from 279.7 million reads (Illumina and 454) generated for the 56 peach accessions multiplexed among 12 pools (Table 1). Excluding one sequencing run for ‘Lovell’ that generated an unusually low number of reads, the total number of reads sequenced using the Illumina platform averaged 2.16× coverage per accession and ranged from ∼0.56 to 19.2 million reads in ‘Tabacchiera’ and ‘Sahua Hong Pantao’, respectively. The total number of reads per accession sequenced using the 454 platform averaged 0.22× coverage and ranged from 0.145 to 0.198 million reads in ‘Sweet Cap’ and ‘Big Top’, respectively. The number of SNPs identified after Stage 1 detection was 943,549 for pools 1–5 and 57,933 for pools 6–11 (Figure 1). When the same filtering parameters as those used for pool 6–11 were reapplied to pools 6–12, the number of SNPs increased to 78,805.

When the SNP calls were compared among the three pairs of independently sequenced accessions, the majority of the positions were not covered by at least one of the two datasets and corresponded to 297,078, 301,617 and 309,027 positions in ‘Elberta’, ‘Big Top’ and ‘O'Henry’, respectively. Thus, the SNP positions that could be compared among these accessions ranged from 3.75% and 4% of the SNP calls in ‘O'Henry’ and ‘Big Top’ (sequenced by Illumina and 454) to 7.48% in Illumina-resequenced ‘Elberta’. In these positions, most of the SNPs had the same genotype (51.2%, 72.4% and 79.9% of reads in ‘Elberta’, ‘Big Top’ and ‘O'Henry’, respectively) while a negligible number of positions did not share any allele (0.05% of positions in ‘Big Top’ to 0.6% in ‘Elberta’ and ‘O'Henry’).

### SNP validation

Several SNP characteristics were associated with performance differences in the GoldenGate® assay (Table 2). Exonic and intronic SNPs were the most successful, with approximately 75% of polymorphisms verified; in contrast, intergenic SNPs were the worst performers, with only a third being polymorphic and a third failing (Table 2, Figure S2). Although MAF observed from sequencing of the detection panel (n = 23) and MAF observed after GoldenGate® genotyping of the validation panel (n = 119) were not well correlated (R = 0.12), the higher the detection panel MAF of a SNP, especially >30%, the more likely its validation panel MAF was >10%. Yet SNPs with a detection panel MAF of <20% were more likely than higher MAF SNPs to convert to a validation panel MAF of <10% without a higher-than-average rate of failure or monomorphism (Table 2). Of the 74 SNPs evenly distributed over the genome, those on LG6, LG1, and LG3 had the highest conversion to polymorphism yet did not have a higher-than-average proportion of exonic and intronic SNPs (Table 2, Figure S2). LG1 and LG6 also had the highest SNP representation because they were the longest in genetic length. LG8 gave a high proportion of polymorphic SNPs with low MAF (<10% in the validation panel). LG2 had a very high failure rate yet only an average proportion of exonic and intronic SNPs (Table 2, Figure S2). Accession-specific SNPs had a close to average performance for rate of failure, monomorphism, and polymorphism, but provided a higher-than-average proportion of low MAF SNPs observed in the validation panel. No SNPs designated as accession-specific in the detection panel remained accession-specific after genotyping the validation panel, although those developed from the almond accession ‘Nonpareil’ were obviously indicative of almond introgression in the validation panel. SNPs targeted to the *F-M* locus also provided a high proportion of low-MAF SNPs, but had a high failure and monomorphism rate as did the SNPs targeted to other trait loci (Table 2). All trait locus-targeted SNPs were associated with a very high proportion of intergenic SNPs (Figure S2). Overall, SNP failure was similar across all parameters except for a high level for SNPs that were on LG2, targeted to trait loci, or intergenic. Monomorphism was highest for SNPs with a detection panel MAF of 21–30%, trait locus-targeted, or those that were intergenic (Table 2).

**Table 2:** Validation outcomes for 96 SNPs with the GoldenGate® assay.

SNP parameter	Total	Proportion of SNPs					
		Failed	Mono-morphic	Poly-morphic	MAF (validation panel)		
					<5%	5–10%	>10%
Evenly spaced	74	0.22	0.14	0.65	0.01	0.04	0.59
*F-M* locus	14	0.29	*0.36*	*0.36*	**0.07**	**0.14**	*0.14*
Other trait loci	8	*0.38*	*0.38*	*0.25*	0.00	0.00	*0.25*
Accession-specific	19	0.26	0.11	0.63	**0.11**	**0.11**	0.42
Genomic location:							
LG1	13	0.08	0.15	**0.77**	0.00	0.00	**0.77**
LG2	7	*0.71*	0.00	*0.29*	0.00	0.00	*0.29*
LG3	7	0.14	0.14	**0.71**	0.00	0.00	**0.71**
LG4	9	0.33	0.11	0.56	0.00	0.11	0.44
LG5	7	0.14	0.29	0.57	0.00	0.00	0.57
LG6	12	0.00	0.17	**0.83**	0.00	0.00	**0.83**
LG7	10	0.30	0.10	0.60	0.00	0.00	0.60
LG8	9	0.22	0.11	0.67	**0.11**	**0.22**	*0.33*
Genic location:							
Exonic	30	0.13	0.10	**0.77**	0.03	0.03	**0.70**
Intronic	19	0.21	0.05	**0.74**	0.00	0.05	**0.68**
UTR	16	0.25	0.25	0.50	0.00	0.00	0.50
Intergenic	31	*0.35*	*0.32*	*0.32*	0.03	0.10	0.19
ADT score:							
<0.2	2	*1.00*	0.00	0.00	0.00	0.00	0.00
0.2–0.4	4	*1.00*	0.00	0.00	0.00	0.00	0.00
0.4–0.5	2	0.00	*0.50*	0.50	0.00	0.00	0.50
0.5–0.6	1	0.00	0.00	0.00	0.00	0.00	1.00
0.6–0.7	8	*0.38*	0.13	0.50	0.00	**0.13**	**0.38**
0.7–0.8	10	0.20	0.30	0.50	**0.10**	0.00	0.40
0.8–0.9	18	0.17	0.22	0.61	0.06	0.00	**0.56**
>0.90	54	0.22	0.17	0.61	0.00	0.07	**0.54**
MAF (detection panel):							
1–10%	8	0.25	0.25	0.50	0.00	**0.13**	0.38
11–20%	20	0.25	0.15	0.60	**0.10**	**0.15**	0.35
21–30%	9	0.00	*0.56*	0.44	0.00	0.00	0.44
31–40%	25	0.24	0.29	0.56	0.00	0.04	**0.52**
41–50%	34	0.20	0.09	0.62	0.00	0.00	**0.62**
Total	96	0.24	0.19	0.57	0.02	0.05	0.50

Observed minor allele frequency (MAF) of polymorphic SNPs among 23 accessions of the detection panel and 119 non-seedling accessions of the validation panel are indicated. Values in **bold** represent considerably better than average SNP performance (e.g., high polymorphism), while values in *italics* are worse than average (high failure and monomorphism).

Of the 32 validation SNPs that were polymorphic in the interspecific T×E bin-mapping set, 31 had joint genotypes corresponding to their expected genomic region. The presence of null alleles was evident for 14 of these polymorphic SNPs. One intergenic SNP targeted to the *F-M* locus on LG4 (bin 4:63; Scaffold_4:22274908) resulted in polymorphism (MAF 23%) with a joint genotype that unexpectedly placed it on LG2 (bin 2:13).

Of the 55 SNPs polymorphic in the entire validation panel, 52 (95%) were polymorphic in at least one of the five six-seedling breeding progenies. One SNP was polymorphic in all five progenies, 10 in four progenies, 17 in three progenies, 10 in two, and 14 in just one progeny. Proportions of the 55 polymorphic SNPs that were polymorphic within individual progenies were 58%, 40%, 53%, 44%, and 42% from Arkansas (University of Arkansas Fruit Research Station, Clarksville), California (National Clonal Germplasm Repository for Fruit and Nut Crops at Davis, CA), California (UC Davis Wolfskill Orchard at Winters, CA), South Carolina (Clemson University Musser Fruit Research Station, Clemson, SC), and Texas (Texas A&M University-College Station Research farm), respectively. These proportions were lower bounds, given that the presence of segregating null alleles was not considered for these progenies.

### SNP final choice

**Figure 2:**
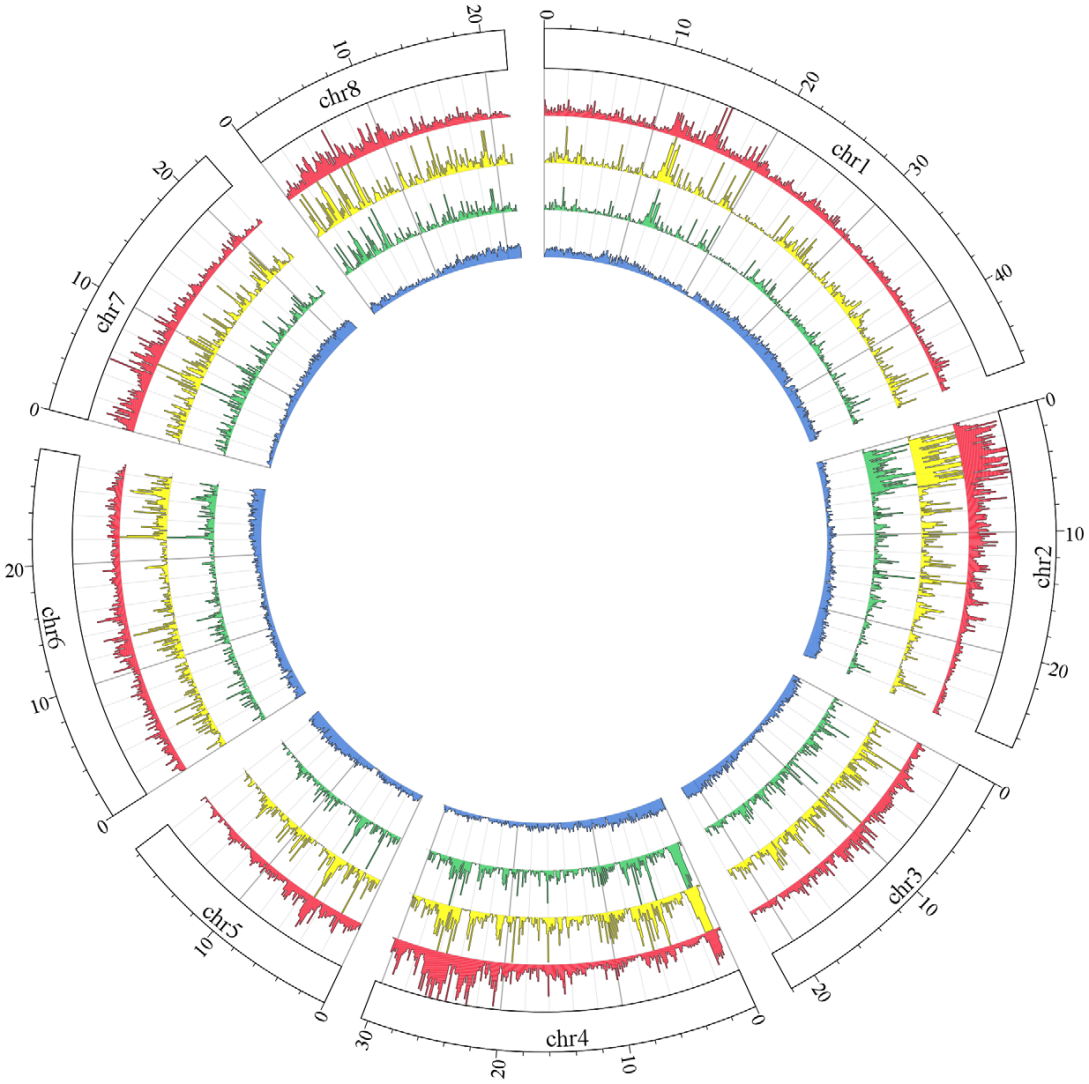
Distribution of SNPs along the Peach v1.0 pseudomolecules. All tracks are plotted in 100 kb windows; inner blue track represents the frequency of coding DNA sequence CDS; y axis ranges from 0 to 100%. Red, yellow and green tracks represent, respectively, absolute number of SNPs discovered within pool 1–5, 40,789 Stage 2 SNPs in exons, and 9,000 SNPs chosen for the array; values in the y axes are capped at 2000, 100, and 30, respectively.

Stage 2 filtering reduced the one million available SNPs by 96%. Restricting the SNPs to Infinium® II types eliminated 18% of Stage 1 SNPs from consideration, the thresholds of ADT≥0.9 and supporting evidence from at least five accessions eliminated 50% of Stage 1 SNPs, and restriction to genic regions cut an additional 23%. Considering only exonic SNPs more than halved the remaining 86,360 to 41,800. Finally, 1006 SNPs located on peach genome scaffolds as yet unassigned to one of the eight chromosomes of this crop were discarded, resulting in 40,794 Stage 2 SNPs (Figure 1, Table 3). The distribution of Stage 2 SNPs varied among and within the eight peach chromosomes (Figure 2, Table 3).

**Table 3:** Chromosome distribution and performance of SNPs on the IPSC peach 9 K SNP array v1.

Chromosome	Stage 2 SNPs	SNPs on peach array	SNPs polymorphic on the peach array	Rate of Polymorphism (%)	Distance between SNPs		
					Average gap (kb)	Largest gap (kb)	No. of gaps >150 kb
1	5573	1114	919	82.5	42.0 (50.9)	672.9 (1254.0)	78 (78)
2	7205	1396	1193	85.5	19.2 (22.3)	521.6 (531.5)	30 (31)
3	4031	811	680	83.8	27.1 (32.3)	393.1 (398.0)	24 (26)
4	8149	1619	1391	85.9	18.6 (21.6)	500.6 (661.2)	26 (25*)
5	2692	546	459	84.1	33.3 (39.3)	915.8 (915.8)	19 (23)
6	4674	933	802	86.0	30.6 (35.6)	515.2 (515.2)	27 (30)
7	3943	793	668	84.2	28.6 (33.3)	484.8 (564.9)	27 (29)
8	4527	913	743	81.4	23.7 (28.8)	491.4 (634.5)	21 (22)
Total on chromosomes	40794	8125	6855	84.4	26.7 (31.5)	915.8 (1254.0)	252 (264)
*Unanchored scaffolds*	*1006*	*19*	*14*	*73.7*	-	-	-
Total	41800	8144	6869	84.3	-	-	-

* Two >150 Kb gaps collapsed into one (∼600 Kb) after removal of monomorphic SNPs.

Chromosomes and distances refer to pseudomolecules of the whole genome Peach v1.0 assembly. Gaps in brackets refer to those obtained when only polymorphic SNPs are considered.

A total of 649 pre-validated SNPs considered for inclusion in the final array were reduced to 387 by the filtering parameters used for these SNPs. Of the 55 polymorphic SNPs from the GoldenGate® validation assay, 45 were included. Of the 453 peach RosCOS SNPs considered, 225 were included in the final array. SNPs requested for inclusion from the international community were 108 from the DRUPOMICS project (Verde I., unpublished data) and 33 from the University of Chile (Silva H., unpublished data), of which 87 and 30 were included, respectively. Nineteen of the 387 pre-validated SNPs were located on unanchored scaffolds of the Peach v1.0 assembly, four of which were RosCOS SNPs. Inclusion of the 387 pre-validated SNPs left 8,613 positions available in the final array for SNPs distributed over the eight peach chromosomes.

**Figure 3:**
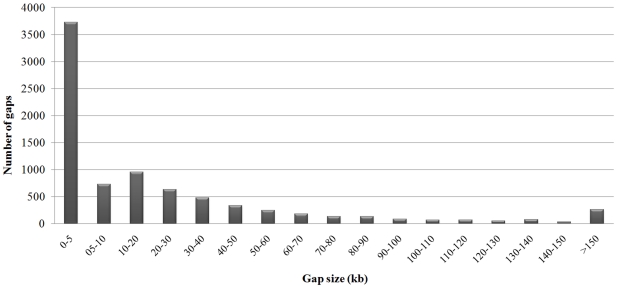
Frequency distribution of size of gaps between SNPs included on the IPSC peach 9 K SNP array v1. Gap sizes were based on SNP physical locations in the Peach v1.0 assembly.

The IPSC peach 9 K SNP array v1 achieved an average spacing of 26.7 kb between SNPs (Table 3). Distribution of SNPs along the Peach v1.0 pseudomolecules varied according to the number of Stage 2 SNPs observed throughout the genome (Figure 2). The largest average gap between successive SNPs (42.0 kb) occurred on chromosome 1 and the smallest on chromosome 4 (18.6 kb). A total of 252 gaps were larger than 150 kb and the largest single gap was 915.8 kb on chromosome 5 (Table 3), but the vast majority of gaps were less than 5 kb (Figure 3). For most of the regions with large gaps there were simply no SNPs available to reduce the gap size considerably. However some of the gaps were caused by the loss of 856 SNPs that occurred during the manufacturing of the array.

### SNP array evaluation

**Figure 4:**
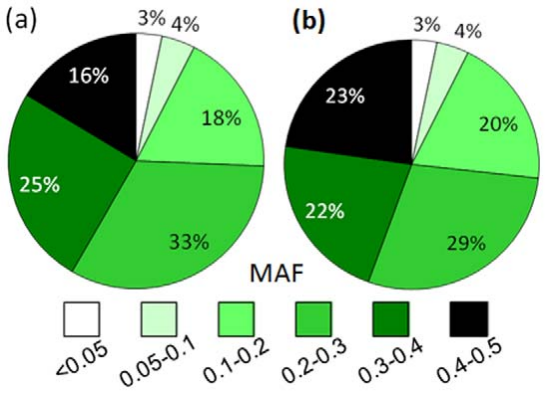
Distribution of minor allele frequencies (MAF) in two independent germplasm sets. A. EU evaluation panel (n = 232); B. US evaluation panel (n = 115; cultivars and advanced selections only).

**Figure 5:**
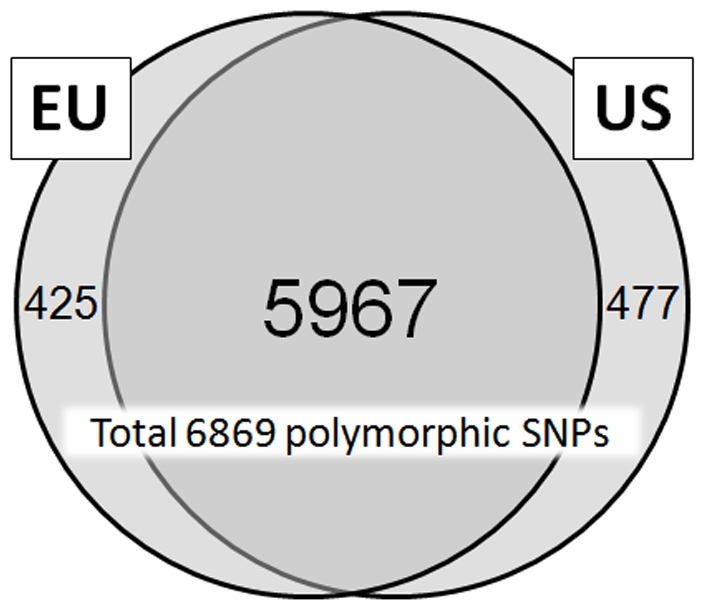
Polymorphic SNPs detected in EU (n = 232) and US (n = 477) evaluation panels from genome scans with the IPSC peach 9 K SNP array v1.

Of the 9,000 candidate SNPs, 8,144 remained on the array after Illumina technical dropout (loss during array manufacturing). Of these, 8,125 were located on the first eight pseudomolecules of the peach genome. The evaluation of IPSC peach 9 K SNP array v1, performed in Europe and U.S., revealed no significant difference in the number of polymorphic, monomorphic and failed SNPs between peach-only samples and samples with interspecific backgrounds (data not shown). The only difference was observed for SNPs located in RosCOS, with only 2% of them being polymorphic in peach-only samples versus 6% in all samples, including those with interspecific ancestry. Moreover, independent evaluation of the IPSC peach 9 K SNP array v1 using EU and US accession panels revealed almost identical results for the numbers of polymorphic, monomorphic and failed SNPs. Genome scans performed with the IPSC peach 9 K SNP array v1 on the EU and US evaluation panels resulted in a SNP polymorphism rate of 70% for each panel and a failure rate of only 5% for each (Table S3). Of the polymorphic SNPs, 92% were observed with a MAF>0.10 for each panel, and 74% and the 69% had a MAF>0.20 in the EU and US evaluation panels, respectively (Table S3). When the US evaluation panel was reduced to only cultivars and advanced selections, the proportion of MAF>0.20 (73%) was almost identical to that observed in the EU evaluation panel. Proportions of SNPs in each MAF range were similar between the two evaluation panels regardless of the type of the material analyzed (Figure 4). In addition to 5,967 SNPs (73%) polymorphic in both evaluation panels, 425 (5%) and 477 (6%) were polymorphic only in the EU and US panels, respectively (Figure 5).

**Figure 6:**
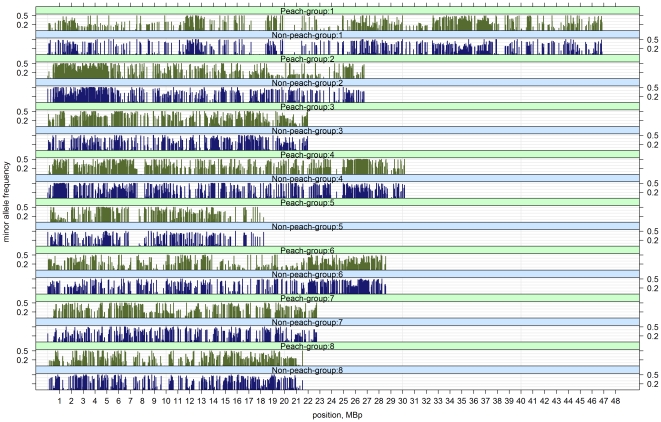
Distribution and physical spacing of polymorphic SNPs across the eight peach chromosomes and comparison of SNP minor allele frequencies between peach and non-peach samples, including almond, and peach and almond wild relatives, in US data set. Data set comprises cultivars and advanced selections only. The coefficient of regression (r) between the MAF in peach and non peach set is 0.437.

The IPSC peach 9 K SNP array v1 achieved a total of 6,869 polymorphic SNPs across the 709 accessions scanned in the two panels combined (Table 3), representing 84.3% of SNPs present on the array. These polymorphic markers provided an average spacing of 31.5 kb across the peach genome, which was consistent among the chromosomes. Fourteen SNPs out of the nineteen localized on the unanchored scaffolds of Peach v1.0 assembly were polymorphic. They provide useful coverage for the unmapped fraction of the Peach v1.0 assembly by helping to reduce gaps. The largest gaps between polymorphic SNPs were on chromosome 1 (1,254 kb). Physical positions and MAFs of polymorphic SNPs were compared for the US peach and non-peach cultivars and selections (Figure 6). The coefficient of regression (r) between MAF in “peach cultivars and selections" and in “non-peach cultivars and selections" within the US panel was 0.437. Predicted SNPs in RosCOS loci were mostly monomorphic, with only 14 being polymorphic.

## Discussion

### SNP detection

The large difference in the number of SNPs identified between pools 1–5 and pools 6–11 is not surprising given the much higher sequencing coverage of pools 1–5 (84.8×) than 6–11 (25.1×) (Table 1) and the greater genetic diversity expected in pools 1–5. Updates in software and hardware for the Illumina Genome Analyzer during the study were major reasons for the difference in coverage between the two main pool groups. Accessions in pools 6–11 were sequenced in February 2010 with GAIIx technology, when the expected number of reads was 17–21 million reads per lane. Accessions in pools 1–5 were sequenced between May and June 2010 with an Illumina GAIIx, when a normal run at IGA was producing about 60 million reads per lane. This higher coverage in pools 1–5 and the SNP validation results based on pools 6–11 prompted some adjustments to the filtering adopted for SNP prediction in pools 1–5. Modern western peach germplasm has a narrow genetic base [15]. In addition to including cultivars of modern U.S. and European breeding programs, pools 1–5 also included landraces from around the world (China, Italy, Japan, Spain, Pakistan, and Brazil). In contrast, the set of accessions of pools 6–11 was less diverse, comprising mostly founders of importance to modern U.S. peach breeding programs.

The low overall percentage of SNPs with matching genotypes in the three independently sequenced pairs of accessions is caused by low sequence coverage targeted here. The sequences generated in this project that were not included in the IPSC peach 9 K SNP array v1 are a valuable resource for peach and other *Prunus* species as they can be searched for additional sequence variation in regions underlying traits of economic importance.

### SNP validation

SNP validation was a critical step for empirically determining appropriate parameters for prioritizing detected SNPs, improving the SNP detection for pools 1–5 that was conducted after SNP detection for pools 6–11, and to choose from among approximately one million SNPs just 9,000 used in the final array design. The validity of the GoldenGate® genotyping results for extrapolation of parameter thresholds to all detected SNPs was supported by the correct genomic location assignment of polymorphic SNPs by bin-mapping. Our observation that conversion of NGS-detected SNPs to true SNPs was more reliable for polymorphisms located in predicted exons and introns was consistent with observations in apple [6]. Better performance of genic SNPs may be due to a lower level of undetected sequence variation in SNP-flanking regions, as opposed to reduced sequence conservation in UTRs and intergenic space, as argued by Chagné et al. [6]. If so, deeper sequencing of detection panel accessions to identify a larger proportion of the polymorphisms would enable avoidance of SNPs with polymorphisms in flanking probe sequences and hence increase the probability of successful SNP marker development. The low depth of sequencing of individual accessions in the detection panel is the probable reason for the lack of significant correlation between detection panel MAF and validation panel MAF. However, detection panel MAF was still a useful parameter for developing rare (<10%) or common (>10%) SNPs. Rare SNPs would be useful for detecting unique haplotypes in a germplasm set, although they would be uninformative for most individuals. Common SNPs would be useful for detecting the majority of genetic variation in a germplasm set and, with a high degree of saturation of any given genomic region, may detect most or all unique haplotypes and thus overcome a lack of rare lineage-specific SNPs. The accession specificity of a SNP in the detection panel was found to be a redundant criterion for developing rare lineage-specific SNPs, and in any case, the relatively small number of accessions in the detection panel and low sequencing coverage of each negated true specificity.

The reason for differential performance of SNPs among some LGs is not clear. In the development of a genome-wide array, it is not advisable to avoid certain LGs or to saturate others just to increase the overall proportion of successful SNPs. These differences were not maintained in the final array (Table 3) and hence probably represent sampling error in the relatively small set of validation SNPs.

Although only about half of the successful SNPs segregated in any of the four individual F_1_ populations included in the validation assay, together these four populations captured most (95%) of the polymorphisms. This observation highlights the importance of utilizing diverse target germplasm rather than a narrow subset of the target genepool, such as a single population, for SNP evaluation and assessment of array informativeness.

### SNP final choice

The SNPs identified within this internationally coordinated effort were subjected to stringent filtering criteria to maximize the efficacy of the IPSC peach 9 K SNP array v1. Given that most candidate SNPs were essentially anonymous, Infinium® I SNPs were avoided because these require two bead types and thus use two SNP slots of the assay, unlike the single bead type for Infinium® II SNPs. Infinium® II SNPs have also been reported to perform better (92% vs. 85%) [3]. Furthermore, Infinium® II SNPs were the most abundant class detected in our study: 82% of the SNPs detected were of this type. SNPs with an ADT score higher than 0.9 were used to increase the probability of SNP success as observed in the validation assay. This Illumina design score reflects the predicted ability of the SNP-flanking sequences to provide a successful assay [5]. The availability of a large number of SNPs allowed us to employ a stringent threshold that was higher than that used in the recently released Illumina Beadchips for apple, pig and chicken, which used ADT thresholds of 0.7, 0.8 and 0.6, respectively [6], [4], [5].

An important aspect we took into consideration in choosing the SNPs for the array was their genomic context. The large number of SNPs discovered in this study allowed us to select 9,000 exonic markers to be included in the array. The high level of transferability of transcriptomic markers across *Prunus* species [27] makes these exonic SNPs useful tools even for related non-peach species such as almond. We also expected exonic SNPs to be more commonly associated with causative mutations underlying phenotypic differences than intergenic SNPs, because of their potential to alter protein sequences.

Finally, another important criterion for a whole genome genotyping assay is to have a uniform distribution of the SNPs across the genome, as this greatly facilitates finding associations between markers and phenotypes. The SNPs on our array cover most of the peach genome with markers well distributed over all chromosomes. The average gap size across the genome achieved was 26.7 kb and increases to 31.5 kb when only polymorphic SNPs are considered. The average ratio of genetic to physical distance in peach is about 440 kb/cM, which was obtained by comparing the Peach v1.0 assembly with an updated version of the *Prunus* reference map [23] (IPGI unpublished results), and this gives an average of 13.3 polymorphic SNPs per cM for our array. Such resolution provides unprecedented power to dissect the peach genome for pinpointing QTLs and determining genetic relatedness. However, SNPs on the array developed here were not evenly spaced across the genome physically or genetically, and a few regions remained with gaps up to 915.8 kb (1,254 kb when considering only polymorphic markers). As the SNP spacing on the array was determined from the density of SNPs detected across the genome and was randomly but proportionally reduced by filtering and non-polymorphism, we expect that the largest gaps exist in regions of low polymorphism (low informativeness) in this crop. Even the largest gap corresponds to a genetic distance of about 2.8 cM, which is still powerful for QTL and relatedness studies. LD decay in peach was estimated at about 13–15 cM [14], so even the regions with large gaps have a density of markers at least 5-fold higher than estimated to be needed to perform optimal GWAS in peach.

### SNP array evaluation

In summary, the total of 6,869 SNPs on the IPSC peach 9 K SNP array v1 verified as polymorphic through extensive empirical evaluation represent an excellent source of markers for studies in genetic relatedness, genetic mapping, and dissecting the genetic architecture of complex agricultural traits. The SNPs included in the array were successfully used for genotyping 709 accessions in two independent evaluation panels. The majority (86.9%) of these markers were polymorphic in both experiments (Figure 5) indicating that the array contains a very low number of false positive SNPs. The comparison (Figure 6) between peach and non-peach accessions showed a common pattern in MAF distribution and large common gaps. The correlation (r = 0.437) between MAF calculated within peach vs. non peach cultivars and selections shows that the array can be efficiently used in peach related species as well. The most notable difference in MAF for the polymorphic SNPs between the peach and non-peach selections was on chromosome 2 around 19 Mbp, where the non-peach group had higher MAF. The common gaps on chromosome 1 (∼21 Mbp), chromosome 2 (∼8.5 Mbp), chromosome 4 (∼24.5 Mbp), chromosome 5 (∼7 Mbp), and chromosome 8 (∼10 Mbp), may represent putative centromeric regions (S. Scalabrin, personal communication).

### Conclusion

The SNP array described here will foster genetic studies in the stone fruits and will help bridge the gap between genomics and breeding activities because breeding germplasm was the basis of detected SNPs and SNP choices of the final array. The IPSC peach 9 K SNP array v1 is commercially available and we expect that it will be used worldwide for genetic studies in peach and related stone fruit and nut species.

## Supporting Information

Figure S1 **Characteristics of 96 SNPs used in a validation assay to test various parameters.**
(TIF)

Figure S2 **Performance of a 96-SNP subset according to genic and genomic location, using the GoldenGate® assay on 160 accessions of the validation panel.**
(TIF)

Table S1 **Accessions of the validation panel used for a GoldenGate® assay of 96 SNPs.** (a) 124 accessions (cultivars, selections, and miscellaneous seedlings). (b) Six full sib progenies of 6 seedlings each. “-" = unknown parent.
(DOCX)

Table S2 **Accessions in two independent evaluation panels used for evaluation of the OPSC peach 9 K SNP array v1.** (a) 232 cultivars and selections from the European Union (EU panel). (b) 115 cultivars and selections from the USA (partial US panel) (c) 362 seedlings of breeding progenies from the USA (remainder of US panel). “-" = unknown parent.
(DOCX)

Table S3 **Performance of the IPSC peach 9 K SNP array v1 on (a) EU (n = 232) and (b) US (n = 477) evaluation panels.**
(DOCX)
