# Correction: Development and Evaluation of a 9K SNP Array for Peach by Internationally Coordinated SNP Detection and Validation in Breeding Germplasm

**DOI:** 10.1371/annotation/33f1ba92-c304-4757-91aa-555de64a0768

**Published:** 2012-06-21

**Authors:** Ignazio Verde, Nahla Bassil, Simone Scalabrin, Barbara Gilmore, Cynthia T. Lawley, Ksenija Gasic, Diego Micheletti, Umesh R. Rosyara, Federica Cattonaro, Elisa Vendramin, Dorrie Main, Valeria Aramini, Andrea L. Blas, Todd C. Mockler, Douglas W. Bryant, Larry Wilhelm, Michela Troggio, Bryon Sosinski, Maria José Aranzana, Pere Arús, Amy Iezzoni, Michele Morgante, Cameron Peace

There was an error in Affiliation 1 for authors Ignazio Verde, Elisa Vendramin, and Valeria Aramini. Affiliation 1 should be: CRA - Fruit Tree Research Center, Rome, Italy, 

