# Supplementary figures and images for: Development and Evaluation of a 9K SNP Array for Peach by Internationally Coordinated SNP Detection and Validation in Breeding Germplasm
Source: PLoS One. 2012 Apr 20;7(4):e35668. doi: 10.1371/journal.pone.0035668 (PMC3334984; doi:10.1371/journal.pone.0035668)

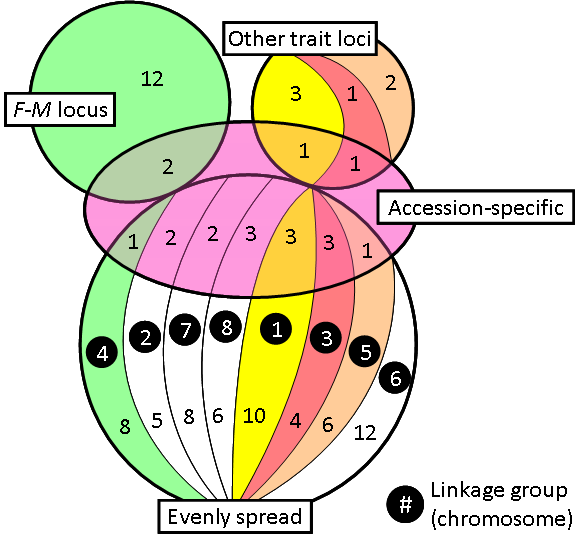

Supplement: Figure S1 — Characteristics of 96 SNPs used in a validation assay to test various parameters. (TIF) [file pone.0035668.s001.tif]

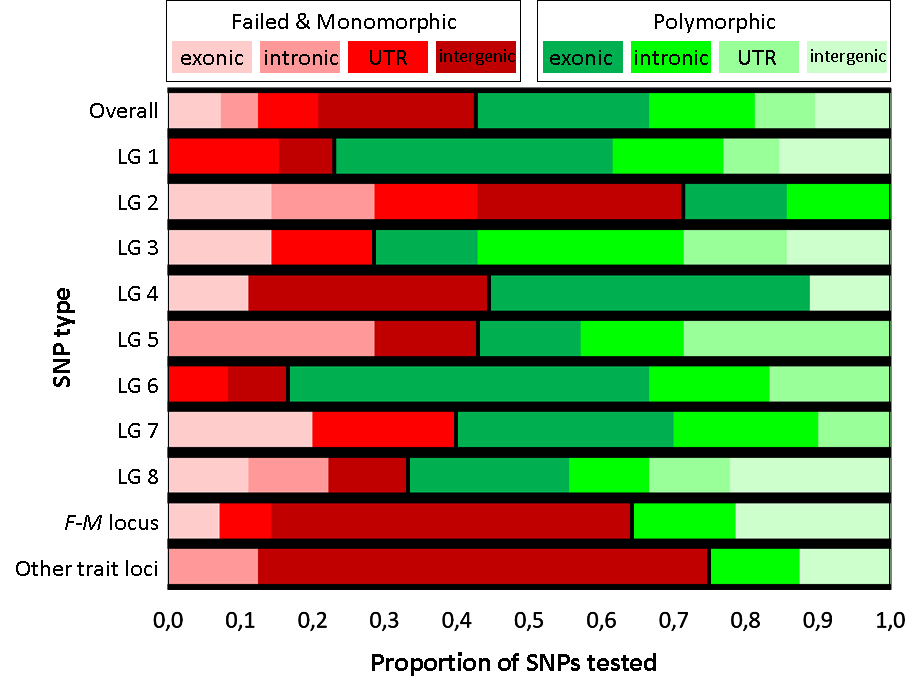

Supplement: Figure S2 — Performance of a 96-SNP subset according to genic and genomic location, using the GoldenGate® assay on 160 accessions of the validation panel. (TIF) [file pone.0035668.s002.tif]
