# Supplementary material for: Development and Evaluation of a 9K SNP Array for Peach by Internationally Coordinated SNP Detection and Validation in Breeding Germplasm
Source: PLoS One. 2012 Apr 20;7(4):e35668. doi: 10.1371/journal.pone.0035668 (PMC3334984; doi:10.1371/journal.pone.0035668)
Supplement: Table S1 — Accessions of the validation panel used for a GoldenGate® assay of 96 SNPs. (a) 124 accessions (cultivars, selections, and miscellaneous seedlings). (b) Six full sib progenies of 6 seedlings each. “-" = unknown parent. (DOCX) [file pone.0035668.s003.docx]

**Table S1:** Accessions of the validation panel used for a GoldenGate® assay of 96 SNPs. (a) 124 accessions (cultivars, selections, and miscellaneous seedlings). (b) Six full sib progenies of 6 seedlings each. “-” = unknown parent.

(a)

| Accession | Mother | Father | Type |
| --- | --- | --- | --- |
| ‘Admiral Dewey’ | - | - | cultivar |
| ‘Andross’ | ‘Fortuna’ | Dix,5A-1 | cultivar |
| ‘Babcock’ | ‘Strawberry’ x ‘Peento’ | ‘Strawberry’ x ‘Peento’ | cultivar |
| ‘Blazeprince’ | BY81P2840 | - | cultivar |
| ‘Bolinha’ | - | - | cultivar |
| ‘Bowen’ | - | - | cultivar |
| ‘Bradley’ | A-190 | A-178 | cultivar |
| ‘Carmen’ | ‘Elberta’ | ‘Family Favorite’ | cultivar |
| ‘Carson’ | ‘Leader’ | ‘Maxine’ | cultivar |
| ‘China Pearl’ | ‘Contender’ | PI 134401 | cultivar |
| ‘Chinese Cling’ | - | - | cultivar |
| ‘Clayton’ | ‘Pekin’ | ‘Candor’ | cultivar |
| ‘Conserva 458’ | - | - | cultivar |
| ‘Contender’ | ‘Winblo’ | NC64 | cultivar |
| ‘Crimson Lady’ | ‘Red Diamond’ | ‘Springcrest’ | cultivar |
| ‘Diamante’ | - | - | cultivar |
| ‘Dixon’ | ‘Australian Muir’ | ‘Orange Cling’ | cultivar |
| ‘Dr. Davis’ | D25-9E | G40-5E | cultivar |
| ‘Early Crawford’ | - | - | cultivar |
| ‘Earlygold’ | - | - | cultivar |
| ‘Elberta’ | ‘Chinese Cling’ | ‘Early Crawford’ | cultivar |
| ‘Everts’ | Dix 22A-5 | Dix 5A-1 | cultivar |
| ‘Flordaprince’ | Fla. 2-7 | ‘Maravilha’ | cultivar |
| ‘Georgia Belle’ | ‘Chinese Cling’ | - | cultivar |
| ‘Goldprince’ | ‘Loring’ | FV3-257 | cultivar |
| ‘Goodwin’ | ‘Dr. Davis’ | 11-11-37 | cultivar |
| ‘Hakuho’ | ‘Hakuto’ | ‘Tachibana Wasa’ | cultivar |
| ‘Halford’ | - | - | cultivar |
| ‘Hesse’ | ‘Riegels’ | ‘Riegels’ | cultivar |
| ‘J. H. Hale’ | ‘Elberta’ | - | cultivar |
| ‘Kakamas’ | ‘St. Helena’ | - | cultivar |
| ‘Klampt’ | ‘Dixon’ | ‘Wiser’ | cultivar |
| ‘Late Crawford’ | - | - | cultivar |
| ‘Loadel’ | ‘Lovell’ | - | cultivar |
| ‘Lovell’ | - | - | cultivar |
| ‘Mayflower’ | - | - | cultivar |
| ‘Ogawa’ | 90,10-91 | 90,10-91 | cultivar |
| ‘O'Henry’ | ‘Merrill Bonanza’ | - | cultivar |
| ‘Okinawa’ | - | - | cultivar |
| ‘Oldmixon Free’ | ‘Oldmixon Cling’ | - | cultivar |
| ‘Orange Cling’ | - | - | cultivar |
| ‘Peento’ | - | - | cultivar |
| ‘Riegels’ | ‘Jungerman’ | ‘Everts’ | cultivar |
| ‘Rio Oso Gem’ | ‘LateCrawford’ | - | cultivar |
| ‘Ross’ | D30-3E | GH8-14 | cultivar |
| ‘Slappey’ | - | - | cultivar |
| ‘St. John’ | ‘Chinese Cling’ | - | cultivar |
| ‘Tropic Beauty’ | Fla.3-2 | ‘Flordaprince’ | cultivar |
| ‘UF Gold’ | Fla.84-18C | Fla.9-20C | cultivar |
| ‘White County’ | A-392 | A-433 | cultivar |
| ‘Winblo’ | ‘Redskin’ | ‘Redskin’ | cultivar |
| ‘Woltemade’ | ‘Kakamas’ | - | cultivar |
| ‘Yumyeong’ | - | - | cultivar |
| ‘Zin Dai’ | - | - | cultivar |
| ‘Nemaguard’ | - | - | cultivar (hybrid) |
| ‘Nickels’ | CP5-33 | ‘Nemaguard’ | cultivar (hybrid) |
| ‘Vilmos’ | F8,72-33 | - | cultivar (hybrid) |
| ‘Jordanolo’ | ‘Nonpareil’ | ‘Harriott’ | cultivar (almond) |
| ‘Mission’ | - | - | cultivar (almond) |
| ‘Nonpareil’ | - | - | cultivar (almond) |
| 18,8-11 | H,6-55 | - | selection |
| 18,8-23 | H,6-55 | - | selection |
| 40A-17 | - | - | selection |
| 54P455 | ‘Golden Glory’ | ‘Bonanza’ | selection |
| 89,9-82 | H6-55 | ‘Kakamas’ | selection |
| 90,9-116 | ‘Ross’ | R1-1 | selection |
| 91,17-195 | 18,6-33 | 87,13-13 | selection |
| 91,17-262 | 18,6-33 | 87,12-31 | selection |
| 92,14-73 | 89,6-103 | 89,6-103 | selection |
| 96,9-292 | E22-59 | E22-59 | selection |
| 98,2-132 | ‘Pallas’ | F8,1-96 | selection |
| 99,12-155 | ‘Woltemade’ | 91,17-195 | selection |
| 99,15-99 | F8,1-42 | F8,1-42 | selection |
| 99,16-131 | F8,1-121 | F8,1-121 | selection |
| 99,4-123 | 19,4-40 | 93,3-225 | selection |
| 2000,2-16 | ‘Loadel’ | F8,5-166 | selection |
| 2000,2-18 | ‘Loadel’ | F8,5-166 | selection |
| 2000,2-8 | ‘Loadel’ | *P. argentea* | selection |
| 2000,2-9 | ‘Loadel’ | *P. argentea* | selection |
| 2000,3-205 | ‘Andross’ | ‘Mission’ x *P. scoparia* | selection |
| 2000,16-133 | F8,5-159 | F8,5-159 | selection |
| 2000,8-150 | ‘Dr. Davis’ | F8,5-156 | selection |
| 2000,8-153 | ‘Dr. Davis’ | F8,5-156 | selection |
| 2000,8-164 | ‘Dr. Davis’ | F8,5-156 | selection |
| 2000,15-127 | F8,1-71 | F8,1-71 | selection |
| 2000,16-125 | - | - | selection |
| 2001,18-215 | - | - | selection |
| 2001,7-180 | ‘Andross’ | *P. argentea* | selection |
| 2003,1-329 | ‘Dr. Davis’ | *P. mira* #19 | selection |
| 2005,17-05 | ‘Loadel’ | F10C,12-28 | selection |
| 2005,29-95 | 92,14-73 | 92,14-73 | selection |
| A-672 | A-405 | A-419 | selection |
| A-708 | A-434 | A-392 | selection |
| A-716 | - | - | selection |
| A-763 | A-708 | ‘Winblo’ | selection |
| A-765CN | A-699 | A-663 | selection |
| A-772 | A-405 | A-665 | selection |
| A-773 | A-371 | A-604 | selection |
| A-778N | A-405 | A-657 | selection |
| BY01P6245 | ‘Contender’ | Fla.92-2C | selection |
| F10C,20-51 | F8,76-45 | - | selection |
| F8,1-42 | 90,1-4 | 90,1-4 | selection |
| F8,5-147 | 90,10-91 | 90,10-91 | selection |
| F8,5-156 | 90,10-91 | 90,10-91 | selection |
| F8,5-159 | 90,10-91 | 90,10-91 | selection |
| F8,5-166 | 90,10-91 | 90,10-91 | selection |
| F8,5-171 | 90,10-91 | 90,10-91 | selection |
| CAF 2 | P97-14 | Y150-13 | selection |
| CAF 3 | P91-23 | Y142-75 | selection |
| CAF 4 | Y140-77 | Y142-194 | selection |
| D62-193 | NJC83 | ‘Conserva 485’ | selection |
| E22-59 | 18,8-11 | - | selection |
| MB1-73 Hybrid F1 | - | - | selection |
| *P. mira* #19 | - | - | selection |
| *P.persica x P. davidiana* | (peach) | *P. davidiana* | selection |
| S-37 | - | - | selection |
| TX2293-3 | ‘Tropic Beauty’ | ‘Goldprince’ | selection |
| TX2B136 | ‘Hermosillo’ | TXW1293-1 | selection |
| TXW1293-1 | ‘Tropic Beauty’ | ‘Tropic Beauty’ | selection |
| CA 7,12-209 | 2000,16-133 | - | seedling |
| CA 7,12-209 | 2000,16-133 | - | seedling (duplicate of above) |
| CA 7,18-133 | 2000,16-92 | - | seedling |
| CA 7,25-162 | 2000,16-92 | 2000,16-92 | seedling |
| CA 8,29-138 | ‘Dr. Davis’ | 2000,15-119 | seedling |

(b)

| Population | Parentage | No. of seedlings | Seedling numbers |
| --- | --- | --- | --- |
| TxE bin-set | Selfed F1 of ‘Texas’ x ‘Earlygold’ | 6 | 5, 12, 23, 30, 34, 83 |
| AR Pop 1 | ‘White County’ x A-672 | 6 | 1 to 6 |
| CA Pop 5,17 | ‘Carson’ x *P. persica x P. davidiana'* | 6 | 192, 196, 198, 202, 207, 217 |
| CA Pop 8,13 | ‘Loadel’ x 2003,1-329 | 6 | 174, 175, 176, 178, 179, 181 |
| SC Pop 0809 | ‘China Pearl’ x ‘Bolinha’ | 6 | 4, 7, 9, 11, 12, 15 |
| TX Pop 1 | TX2B136 x CAF 4 | 6 | 1 to 6 |
