# Supplementary material for: Development and Evaluation of a 9K SNP Array for Peach by Internationally Coordinated SNP Detection and Validation in Breeding Germplasm
Source: PLoS One. 2012 Apr 20;7(4):e35668. doi: 10.1371/journal.pone.0035668 (PMC3334984; doi:10.1371/journal.pone.0035668)
Supplement: Table S2 — Accessions in two independent evaluation panels used for evaluation of the OPSC peach 9 K SNP array v1. (a) 232 cultivars and selections from the European Union (EU panel). (b) 115 cultivars and selections from the USA (partial US panel) (c) 362 seedlings of breeding progenies from the USA (remainder of US panel). “-" = unknown parent. (DOCX) [file pone.0035668.s004.docx]

**Table S2**: Accessions of two independent evaluation panels used for evaluation of the IPSC peach 9K SNP array v1. (a) 232 cultivars and selections from the European Union (EU panel). (b) 115 cultivars and selections from the USA (partial US panel) (c) 362 seedlings of breeding progenies from the USA (remainder of US panel). “-” = unknown parent.

(a)

| Accession | Mother | Father | Type | Panel |
| --- | --- | --- | --- | --- |
| ‘Ackman Early’ | - | - | cultivar | EU |
| ‘Albatros’ | ‘Princess of Wales’ | - | cultivar | EU |
| ‘Alberge Jaune’ | - | - | cultivar | EU |
| ‘Albertun Brutta’ | - | - | cultivar | EU |
| ‘Aletta’ | - | - | cultivar | EU |
| ‘Allerbe’ | - | - | cultivar | EU |
| ‘Allgold’ | NJ 554367 | G17-5E | cultivar | EU |
| ‘Amarillo De Agosto 1’ | - | - | cultivar | EU |
| ‘Angiolina’ | - | - | cultivar | EU |
| ‘Aniversario’ | - | - | cultivar | EU |
| ‘Aso 43’ | - | - | cultivar | EU |
| ‘Atalanta’ | (‘Bonetti 2’ x ‘Impero’) x ‘Redhaven’ | - | cultivar | EU |
| ‘Avangard’ | - | - | cultivar | EU |
| ‘Aves’ | - | - | cultivar | EU |
| ‘Baekhyang’ | ‘Garden State’ | - | cultivar | EU |
| ‘Baladi 1’ | - | - | cultivar | EU |
| ‘Baldagenais’ | - | - | cultivar | EU |
| ‘Balkonella’ | - | - | cultivar | EU |
| ‘Bambino’ | - | - | cultivar | EU |
| ‘Batiteun De Nus’ | - | - | cultivar | EU |
| ‘Bayue Cui’ | ‘Lu Hua 5’ | ‘Okubo’ | cultivar | EU |
| ‘Beale’ | - | - | cultivar | EU |
| ‘Beigabin’ | - | - | cultivar | EU |
| ‘Bel Fior’ | - | - | cultivar | EU |
| ‘Bella Cartesienne’ | - | - | cultivar | EU |
| ‘Bella Dei Trentini’ | - | - | cultivar | EU |
| ‘Bella Di Fiumicello’ | - | - | cultivar | EU |
| ‘Bella Di Gambettola’ | - | - | cultivar | EU |
| ‘Bella Lucia’ | - | - | cultivar | EU |
| ‘Bianca Russotto’ | - | - | cultivar | EU |
| ‘Bianca Tardiva’ | - | - | cultivar | EU |
| ‘Black Campanin’ | - | - | cultivar | EU |
| ‘Blanca De Jolon’ | - | - | cultivar | EU |
| ‘Bonanza’ | - | - | cutivar | EU |
| ‘Borota’ | - | - | cultivar | EU |
| ‘Br 1’ | ‘Delicioso’ | ‘Panamint’ | cultivar | EU |
| ‘Br 2’ | ‘Aldrighi’ | ‘Cerrito’ | cultivar | EU |
| ‘Br 3’ | ‘Pala’ | - | cultivar | EU |
| ‘Br 6’ | ‘Ambrosio Perret’ | ‘Tapes’ | cultivar | EU |
| ‘Burrona Di Rosano’ | - | - | cultivar | EU |
| ‘Buttapietra’ | - | - | cultivar | EU |
| ‘California’ | P60-38 | ‘Fantasia’ | cultivar | EU |
| ‘Centenaria’ | ‘Docura 2’ | - | cultivar | EU |
| ‘Cerrito’ | ‘Lake City’ x ‘Interludio’ | - | cultivar | EU |
| ‘Champion’ | - | - | cultivar | EU |
| ‘Chiyomaru’ | - | - | cultivar | EU |
| ‘Chui Huang Tao’ | - | - | cultivar | EU |
| ‘City 32-82’ | - | - | cultivar | EU |
| ‘Congres’ | ‘Flacara’ | ‘Splendid’ | cultivar | EU |
| ‘Cotogna Ceccarelli’ | - | - | cultivar | EU |
| ‘Cotogna Del Poggio’ | - | - | cultivar | EU |
| ‘Cotogna Di Rosano’ | - | - | cultivar | EU |
| ‘Cotogna Massima’ | - | - | cultivar | EU |
| ‘Cropopus N. 3’ | - | - | cultivar | EU |
| ‘De Wet’ | NJC18/21 | SN45/3 | cultivar | EU |
| ‘Diego Fuentes’ | - | - | cultivar | EU |
| ‘Diego Sabrina’ | - | - | cultivar | EU |
| ‘Docura’ | ‘Alo-Docura’ | ‘Tutu’ | cultivar | EU |
| ‘Domiziana’ | ‘Southland’ x ‘Pesco Noce 1’ | - | cultivar | EU |
| ‘Early Risinger’ | - | - | cultivar | EU |
| ‘Encore’ | NJ 585414 | ‘Autumnglo’ | cultivar | EU |
| ‘Entella’ | - | - | cultivar | EU |
| ‘Eolia’ | ‘Dixon 1’ | ‘Keimoes’ | cultivar | EU |
| ‘Ermelinda’ | - | - | cultivar | EU |
| ‘Erzsébet’ | - | - | cultivar | EU |
| ‘Escarolita Pec’ | - | - | cultivar | EU |
| ‘Evergreen’ | - | - | cultivar | EU |
| ‘Fior Di Maggio’ | - | - | cultivar | EU |
| ‘Fior Di Novembre’ | - | - | cultivar | EU |
| ‘Fiorenza’ | - | - | cultivar | EU |
| ‘Ford’ | - | - | cultivar | EU |
| ‘Fruher Rote Ingelheimer’ | - | - | cultivar | EU |
| ‘Frumos De Baneasa’ | - | - | cultivar | EU |
| ‘Galopin’ | - | - | cultivar | EU |
| ‘Gemini’ | - | - | cultivar | EU |
| ‘Georgia Belle’ | ‘Chinese Cling’ | - | cultivar | EU |
| ‘GF 305’ | - | - | cultivar | EU |
| ‘Gialla Moavero’ | - | - | cultivar | EU |
| ‘Gialla Tardiva’ (Forlì) | - | - | cultivar | EU |
| ‘Gialla Tardiva Di Cogo’ | - | - | cultivar | EU |
| ‘Gianni Laura Dolce’ | - | - | cultivar | EU |
| ‘Giulia Settembrina’ | ‘J.H. Hale’ | - | cultivar | EU |
| ‘Guerriera’ | - | - | cultivar | EU |
| ‘Hamlet’ | ‘Pekin’ | ‘Candor’ | cultivar | EU |
| ‘Higama’ | - | - | cultivar | EU |
| ‘Hitachired’ | 19-18 | ‘Fantasia’ | cultivar | EU |
| ‘Imera’ | - | - | cultivar | EU |
| ‘Ingo Peach’ | - | - | cultivar | EU |
| ‘Jacquotte’ | - | - | cultivar | EU |
| ‘Jin Feng’ | ‘Early Crawford’ | ‘Phillips’ | cultivar | EU |
| ‘Kanto-14’ | ‘Okayama-3’ | ‘Orange Cling’ | cultivar | EU |
| ‘Kanto-5’ | (‘Kinto’ x ‘Tuscan’)-43 | (‘Okayama-3’ x ‘Orange Cling’)-9 | cultivar | EU |
| ‘Kappa 2’ | - | - | cultivar | EU |
| ‘Kisapáthy1’ | - | - | cultivar | EU |
| ‘Kisapáthy2’ | - | - | cultivar | EU |
| ‘Kompolt 1’ | - | - | cultivar | EU |
| ‘Köncsögi Kopasz’ | - | - | cultivar | EU |
| ‘Krümcsangin’ | - | - | cultivar | EU |
| ‘Kweckergood’ | - | - | cultivar | EU |
| ‘La Pecher’ | ‘La Feliciana’ | - | cultivar | EU |
| ‘Larubra’ | - | - | cultivar | EU |
| ‘Lednická Zlutá’ | - | - | cultivar | EU |
| ‘Lord Napier’ | ‘Early Albert’ | - | cultivar | EU |
| ‘Lutea’ | ‘Belle’ x ‘Fogato’ | ‘Peento’ | cultivar | EU |
| ‘Maddalena Reale’ | - | - | cultivar | EU |
| ‘Madonna Di Giugno’ | - | - | cultivar | EU |
| ‘Masahime’ | 21-18 | ‘Akatsuki’ | cultivar | EU |
| ‘Mb Petrini’ | - | - | cultivar | EU |
| ‘Meigni Pantao’ | - | - | cultivar | EU |
| ‘Miorita’ | - | - | cultivar | EU |
| ‘Miraflores’ | - | - | cultivar | EU |
| ‘Missour’ | - | - | cultivar | EU |
| ‘Montclair’ | - | - | cultivar | EU |
| ‘Morellona’ | - | - | cultivar | EU |
| ‘Mountingold’ | PI 35201 | NJ 196 | cultivar | EU |
| ‘Myojo’ | ‘Yamashita’ | ‘Sims’ | cultivar | EU |
| ‘Nett. Asprofrut’ | - | - | cultivar | EU |
| ‘Nett. Russa 598-81’ | - | - | cultivar | EU |
| ‘Nj Weeping’ | - | - | cultivar | EU |
| ‘Nyikitai Lapo’ | - | - | cultivar | EU |
| ‘Nyikitszkij-85’ | - | - | cultivar | EU |
| ‘Okubo’ | - | - | cultivar | EU |
| ‘Otiecestuviennuit’ | - | - | cultivar | EU |
| ‘Ouachita Gold’ | ‘LaGold’ | ‘Redskin’ x ‘Southern Glow’ | cultivar | EU |
| ‘Ouromel 3’ | ‘Tutu’ | ‘Columbina’ | cultivar | EU |
| P-6339 -1- 2N (dh) | ‘J.H. Hale’ x ‘Southland’ | ‘J.H. Hale’ x ‘Southland’ | cultivar | EU |
| ‘Palazzina’ | - | - | cultivar | EU |
| ‘Paola Matteucci’ | - | - | cultivar | EU |
| ‘Pappardone’ | - | - | cultivar | EU |
| ‘Peento’ | - | - | cultivar | EU |
| ‘Pelo Tardivo’ | - | - | cultivar | EU |
| ‘Percoco Bianco Leonforte’ | - | - | cultivar | EU |
| ‘Percoco Di Turi’ | - | - | cultivar | EU |
| ‘Persi De Vin’ | - | - | cultivar | EU |
| ‘Pesca A Cuore Capozzi’ | - | - | cultivar | EU |
| ‘Pesca Ala’ | - | - | cultivar | EU |
| ‘Pesca Marscianese’ | - | - | cultivar | EU |
| ‘Pesco Dei Santi’ | - | - | cultivar | EU |
| ‘Pesco Fiore Rosa’ | - | - | cultivar | EU |
| ‘Pfalzperle’ | - | - | cultivar | EU |
| ‘Piatta Tardiva Morsiani’ | - | - | cultivar | EU |
| ‘Pirovano 510’ | - | - | cultivar | EU |
| ‘Platicarpa Pirovano’ | - | - | cultivar | EU |
| ‘Platicarpa C’ | - | - | cultivar | EU |
| ‘Poppa Di Venere’ | - | - | cultivar | EU |
| ‘Precocinho’ | ‘Diamante’ | - | cultivar | EU |
| ‘Rancho Resistant’ | S-37 | - | cultivar | EU |
| ‘Reginella I’ | - | - | cultivar | EU |
| ‘Rikakusuimitsu’ | ‘Chinese Cling’ | - | cultivar | EU |
| ‘Robidoux’ | - | - | cultivar | EU |
| ‘Rogati 2’ | - | - | cultivar | EU |
| ‘Rojo Di Albesa’ | - | - | cultivar | EU |
| ‘Romamer 2’ | NJ CITY 29-245 | NJRR 48-153 | cultivar | EU |
| ‘Romantica Proni’ | - | - | cultivar | EU |
| ‘Rosalina’ | ‘Real’ x ‘Sunlite’ | - | cultivar | EU |
| ‘Rossa De Cecchi’ | - | - | cultivar | EU |
| ‘Rossa Di Lugo’ | - | - | cultivar | EU |
| ‘Rossone Della Costa’ | - | - | cultivar | EU |
| ‘Rosu Dungat’ | - | - | cultivar | EU |
| ‘Rou Tao’ | - | - | cultivar | EU |
| ‘Royal Lee’ | - | - | cultivar | EU |
| ‘Russian Flat’ | - | - | cultivar | EU |
| ‘Russotto’ | - | - | cultivar | EU |
| ‘S. Michele Giallo’ | - | - | cultivar | EU |
| ‘Salkaja’ | - | - | cultivar | EU |
| ‘San Giorgio’ | - | - | cultivar | EU |
| ‘San Giovanni’ | - | - | cultivar | EU |
| ‘San Gottardo’ | - | - | cultivar | EU |
| ‘San Varano 2’ | - | - | cultivar | EU |
| ‘San Varano 3’ | - | - | cultivar | EU |
| ‘San Vito’ | - | - | cultivar | EU |
| ‘Sanguigna Di Savoia’ | - | - | cultivar | EU |
| ‘Sanguinella’ (Forlì) | - | - | cultivar | EU |
| ‘Sanguinella’ (Roma) | - | - | cultivar | EU |
| ‘Selvaggio Di Canove’ | - | - | cultivar | EU |
| ‘Septembriska’ | - | - | cultivar | EU |
| ‘Serena Baruzzi’ | - | - | cultivar | EU |
| ‘Settembrina Di Bivona’ | - | - | cultivar | EU |
| ‘Shizukured’ | 19-1 | - | cultivar | EU |
| ‘Sicilia 2’ | ‘Peento’ | - | cultivar | EU |
| ‘Somel’ | ‘Ouromel’ x ‘Sunred’ | ‘Ouromel’ x ‘Sunred’ | cultivar | EU |
| ‘Souvenir Nikitski’ | - | - | cultivar | EU |
| ‘Stark Saturn’ | (‘Saturn’ sport) |  | cultivar | EU |
| ‘Swellen Grebel’ | ‘Babcock’ | ‘Early Dawn’ | cultivar | EU |
| ‘Tapodi’ | - | - | cultivar | EU |
| ‘Tardiva Di Ficarazzi’ | - | - | cultivar | EU |
| ‘Tardiva Di Fresnu’ | - | - | cultivar | EU |
| ‘Tardiva Di Renacci’ | - | - | cultivar | EU |
| ‘Tardiva Di S. Vittorino’ | - | - | cultivar | EU |
| ‘Tardiva Goretti’ | - | - | cultivar | EU |
| ‘Tatura Dawn’ | ‘Levis ‘ | ‘Levis’ | cultivar | EU |
| ‘Terzarola Gialla’ | - | - | cultivar | EU |
| ‘Tos-China D'ottobre’ | ‘Pesca della China’ | ‘Poppa di Venere’ | cultivar | EU |
| ‘Trakjska Ranna’ | - | - | cultivar | EU |
| ‘Vaccaro Roccalmunto’ | - | - | cultivar | EU |
| ‘Vecchi 74’ | - | - | cultivar | EU |
| ‘Vérbélű’ | - | - | cultivar | EU |
| ‘Vespignani Sel. 2’ | - | - | cultivar | EU |
| ‘Vigna Verde’ | - | - | cultivar | EU |
| ‘Vinosa H. De Monfort’ | - | - | cultivar | EU |
| ‘Vittorio Emanuele III’ | - | - | cultivar | EU |
| ‘Wolbong Josaeng’ | (‘Kurakata Wase’ sport) |  | cultivar | EU |
| ‘Xavant’ | - | - | cultivar | EU |
| ‘Yoshihime’ | 21-18 | ‘Akatsuki’ | cultivar | EU |
| ‘Zhao Hui’ | ‘Bai Hua’ | ‘Tasubanawase’ | cultivar | EU |
| ‘Zhao Xia’ | ‘Bei Hua’ | ‘Hakkobi’ | cultivar | EU |
| ‘Zhongsan Zaolu’ | ‘Bei Hua’ | ‘Hakkobi’ | cultivar | EU |
| ‘Zingara Nera’ | - | - | cultivar | EU |
| ‘Zsoltij’ | - | - | cultivar | EU |
| ‘Zverdocen’ | - | - | cultivar | EU |
| ‘Garnem’ | *P. dulcis* | ‘Nemared’ | cultivar (hybrid) | EU |
| ‘Titan’ | *P. dulcis* | ‘Nemaguard’ | cultivar (hybrid) | EU |
| 592-81 | - | - | selection | EU |
| 63-15-33 | - | - | selection | EU |
| Cp 88/2 | - | - | selection | EU |
| Fla 82-10 N | - | - | selection | EU |
| M. A 4870 | - | - | selection | EU |
| Mexican Sel P09.30.52 | - | - | selection | EU |
| Mexican Sel P09.30.60 | - | - | selection | EU |
| Mexican Sel P09.30.65 | - | - | selection | EU |
| Mexican Sel P09.30.78 | - | - | selection | EU |
| IF 8431138 | ‘Stark Sunglo’ x IF7131649 | - | selection | EU |
| IF 8431354 | ‘Stark Sunglo’ x IF7131649 | - | selection | EU |
| IF 8810268 | IF 8431354 | - | selection | EU |
| Phn 91-12 | - | - | selection | EU |
| Phn 91-14 | - | - | selection | EU |
| Phn 91-17 | - | - | selection | EU |
| Php 91-05 | - | - | selection | EU |
| Php 91-10 | - | - | selection | EU |
| Sel. Exoascus Resistente Roma | - | - | selection | EU |
| V 33-2 | - | - | selection | EU |
| O.P.G. | *P. persica* | *P. dulcis* | selection (hybrid) | EU |

(b)

| Accession | Mother | Father | Type | Panel |
| --- | --- | --- | --- | --- |
| ‘Admiral Dewey’ | - | - | cultivar | US |
| ‘Andross’ | ‘Fortuna’ | Dix 5A-1 | cultivar | US |
| ‘Arrington’ | A-178 | A-232 | cultivar | US |
| ‘Blazeprince’ | BY81P2840 | - | cultivar | US |
| ‘Bolinha’ | - | - | cultivar | US |
| ‘Bradley’ | A-190 | A-178 | cultivar | US |
| ‘Candor’ | ‘Redhaven’ | ‘Erly Red Fre’ | cultivar | US |
| ‘Carmen’ | ‘Elberta’ | ‘Family Favorite’ | cultivar | US |
| ‘Carolyn G’ | ‘Libbee’ | ‘Lovell’ | cultivar | US |
| ‘Carson’ | ‘Leader’ | ‘Maxine’ | cultivar | US |
| ‘China Pearl’ | ‘Contender’ | PI 134401 | cultivar | US |
| ‘Chinese Cling’ | - | - | cultivar | US |
| ‘Clayton’ | ‘Pekin’ | ‘Candor’ | cultivar | US |
| ‘Conserva 458’ | - | - | cultivar | US |
| ‘Contender’ | ‘Winblo’ | NC64 | cultivar | US |
| ‘Crimson Lady’ | ‘RedDiamond’ | ‘Springcrest’ | cultivar | US |
| ‘Cumberland’ | ‘Georgia Belle’ | ‘Greensboro’ | cultivar | US |
| ‘Diamante’ | - | - | cultivar | US |
| ‘Dixon’ | ‘Australian Muir’ | ‘Orange Cling’ | cultivar | US |
| ‘Dr. Davis’ | D25-9E | G40-5E | cultivar | US |
| ‘Early Crawford’ | - | - | cultivar | US |
| ‘Elberta’ | ‘Chinese Cling’ | ‘Early Crawford’ | cultivar | US |
| ‘Everts’ | Dix 22A-5 | Dix 5A-1 | cultivar | US |
| ‘Flordaprince’ | Fla.2-7 | ‘Maravilha’ | cultivar | US |
| ‘Galaxy’ | P34-106 | D33-1 | cultivar | US |
| ‘Georgia Belle’ | ‘Chinese Cling’ | - | cultivar | US |
| ‘Goldprince’ | ‘Loring’ | FV3-257 | cultivar | US |
| ‘Goodwin’ | ‘Dr. Davis’ | 11-11-37 | cultivar | US |
| ‘Greensboro’ | - | - | cultivar | US |
| ‘Hakuho’ | ‘Hakuto’ | ‘Tachibana Wasa’ | cultivar | US |
| ‘Halford’ | - | - | cultivar | US |
| ‘Hesse’ | ‘Riegels’ | ‘Riegels’ | cultivar | US |
| ‘Hiley’ | - | - | cultivar | US |
| ‘J.H. Hale’ | ‘Elberta’ | - | cultivar | US |
| ‘Jefferson’ | - | - | cultivar | US |
| ‘Kakamas’ | ‘St. Helena’ | - | cultivar | US |
| ‘Klampt’ | ‘Dixon’ | ‘Wiser’ | cultivar | US |
| ‘Late Crawford’ | - | - | cultivar | US |
| ‘Late Ross’ | (‘Ross’ sport) |  | cultivar | US |
| ‘Lilliland’ | ‘Ross’ | R1-1 | cultivar | US |
| ‘Loadel’ | ‘Lovell’ | - | cultivar | US |
| ‘Lola’ | - | - | cultivar | US |
| ‘Loring’ | ‘Frank’ | ‘Halehaven’ | cultivar | US |
| ‘Lovell’ | - | - | cultivar | US |
| ‘Mayfire’ | ‘Armking’ | - | cultivar | US |
| ‘O'Henry’ | ‘Merrill Bonanza’ | - | cultivar | US |
| ‘Oldmixon Free’ | ‘Oldmixon Cling’ | - | cultivar | US |
| ‘Orange Cling’ | - | - | cultivar | US |
| ‘Panamint’ | ‘Babcock’ x ‘Boston’ | ‘Goldmine’ x ‘Rio Oso Gem’ | cultivar | US |
| ‘Peento’ | - | - | cultivar | US |
| ‘Redhaven’ | ‘Halehaven’ | ‘Kalhaven’ | cultivar | US |
| ‘Redskin’ | ‘J.H. Hale’ | ‘Elberta’ | cultivar | US |
| ‘Riegels’ | ‘Jungerman’ | ‘Everts’ | cultivar | US |
| ‘Rio Oso Gem’ | ‘Late Crawford’ | - | cultivar | US |
| ‘Rizzi’ | ‘Everts’ | - | cultivar | US |
| ‘Ross’ | D30-3E | GH8-14 | cultivar | US |
| ‘Saturn’ | ‘Pallas’ | NJ 602903 | cultivar | US |
| ‘Slappey’ | - | - | cultivar | US |
| ‘Springold’ | FV89-14 | ‘Springtime’ | cultivar | US |
| ‘Springprince’ | ‘Springcrest’ | - | cultivar | US |
| ‘Springtime’ | ‘Lukens Honey’ x ‘July Elberta’ | ‘Robin’ | cultivar | US |
| ‘St. John’ | ‘Chinese Cling’ | - | cultivar | US |
| ‘Sunfre’ | P42-81 | P42-91 | cultivar | US |
| ‘Sunhigh’ | ‘J.H. Hale’ | NJ40CS | cultivar | US |
| ‘Tropic Beauty’ | Fla.3-2 | ‘Flordaprince’ | cultivar | US |
| ‘UF Gold’ | Fla.84-18C | Fla.9-20C | cultivar | US |
| ‘Westbrook’ | A-172 | A-176 | cultivar | US |
| ‘White County’ | A-392 | A-433 | cultivar | US |
| ‘White River’ | ‘Loring’ | NJ257 | cultivar | US |
| ‘Winblo’ | ‘Redskin’ | ‘Redskin’ | cultivar | US |
| ‘Woltemade’ | ‘Kakamas’ | - | cultivar | US |
| ‘Yumyeong’ | ‘Yamato-Wase’ | ‘Nunome-Wase’ | cultivar | US |
| ‘ZinDai’ | - | - | cultivar | US |
| ‘Nemaguard’ | - | - | cultivar (hybrid) | US |
| ‘Nickels’ | CP 5 33 | ‘Nemaguard’ | cultivar (hybrid) | US |
| ‘Ogawa’ | 90,10-91 | 90,10-91 | cultivar (hybrid) | US |
| ‘Vilmos’ | (F10C,12-28 veg. propagule) | | cultivar (hybrid) | US |
| ‘Carmel’ | ‘Nonpareil’ | ‘Mission’ | cultivar (almond) | US |
| ‘Jordanolo’ | ‘Nonpareil’ | ‘Harriott’ | cultivar (almond) | US |
| ‘Mission’ | - | - | cultivar (almond) | US |
| ‘Mission BF’ | (‘Mission’ sport) |  | cultivar (almond) | US |
| ‘Nonpareil’ | - | - | cultivar (almond) | US |
| ‘Sonora’ | ‘Nonpareil’ | - | cultivar (almond) | US |
| ‘Stukey 6-27’ | ‘Nonpareil’ | - | cultivar (almond) | US |
| ‘Stukey 6-27H’ | ‘Nonpareil’ | - | cultivar (almond) | US |
| ‘Stukey 6-8’ | ‘Nonpareil’ | - | cultivar (almond) | US |
| ‘Stukey 6-9BF’ | ‘Nonpareil’ | - | cultivar (almond) | US |
| ‘Tardy Nonpareil’ | (‘Nonpareil’ sport) |  | cultivar (almond) | US |
| 2005,16-191 | H-6-55 | 98,13-17 | selection | US |
| 54P455 | ‘Golden Glory’ | ‘Bonanza’ | selection | US |
| BY01P6245 | ‘Contender’ | Fla.92-2C | selection | US |
| CAF 2 | P97-14 | Y150-13 | selection | US |
| CAF 3 | P91-23 | Y142-75 | selection | US |
| CAF 4 | Y140-77 | Y142-194 | selection | US |
| D62-193 | NJC83 | ‘Conserva485’ | selection | US |
| E22-59 | 18,8-11 | - | selection | US |
| *P. mira* 19 | - | - | selection | US |
| S 37 | - | - | selection | US |
| TX2293_3 | ‘Tropic Beauty’ | ‘Goldprince’ | selection | US |
| TX2B136 | ‘Hermosillo’ | TXW1293-1 | selection | US |
| TXW1293_1 | ‘Tropic Beauty’ | ‘Tropic Beauty’ | selection | US |
| 2000,2-8 | ‘Loadel’ | *P. argentea* | selection (hybrid) | US |
| 2000,2-9 | ‘Loadel’ | *P. argentea* | selection (hybrid) | US |
| 2000,3-205 | ‘Andross’ | ‘Mission’ x *P. scoparia* | selection (hybrid) | US |
| 2000,16-133 | F8,5-159 | F8,5-159 | selection (hybrid) | US |
| 2001,7-180 | ‘Andross’ | *P. argentea* | selection (hybrid) | US |
| 2003,1-329 | ‘Dr. Davis’ | *P. mira* 19 | selection (hybrid) | US |
| 2005,19-139 | 19,2-72 | 2000,3-205 | selection (hybrid) | US |
| 91,17-195 | 18,6-33 | 87,13-13 | selection (hybrid) | US |
| 99,12-155 | ‘Woltemade’ | 91,17-195 | selection (hybrid) | US |
| F10C,12-28 | F8,72-33 | - | selection (hybrid) | US |
| F10C,20-51 | F8,76-45 | - | selection (hybrid) | US |
| F8,1-42 | 90,1-4 | 90,1-4 | selection (hybrid) | US |
| F8,5-166 | 90,10-91 | 90,10-91 | selection (hybrid) | US |
| *P. persica x P. davidiana* | (peach) | *P. davidiana* | selection (hybrid) | US |

(c)

| Population | Mother | Father | No. of seedlings | Panel |
| --- | --- | --- | --- | --- |
| CA Pop 5,10 | ‘Dr. Davis’ | D62-193 | 19 | US |
| CA Pop 5,16 | ‘O'Henry’ | ‘O'Henry’ | 2 | US |
| CA Pop 5,17 | ‘Goodwin’ | ‘Vilmos’ | 21 | US |
| CA Pop 8,3 | ‘Loadel’ | ‘Yumyeong’ | 17 | US |
| SC Pop 0804 | ‘Contender’ | BY92P2710 | 20 | US |
| SC Pop 0809 | ‘China Pearl’ | ‘Bolinha’ | 26 | US |
| SC Pop 0814 | ‘Intrepid’ | ‘Blazeprince’ | 3 | US |
| SC Pop 0815 | ‘Intrepid’ | ‘Bolinha’ | 3 | US |
| SC Pop 0817 | ‘O'Henry’ | ‘Cascata 1006’ | 24 | US |
| SC Pop 0821 | BY92P2710 | ‘Bolinha’ | 20 | US |
| SC Pop 0824 | BY86P2609 | BY86P2609 | 23 | US |
| SC Pop 0825 | BY86P2609 | ‘Bradley’ | 23 | US |
| SC Pop 0826 | BY86P2609 | ‘WhiteRiver’ | 12 | US |
| SC Pop 0836 | ‘Contender’ | ‘Bolinha’ | 7 | US |
| SC Pop B | BY02P4019 | BY02P4019 | 25 | US |
| TX Pop 2 | TX2B136 | CAF 2 | 22 | US |
| CA Pop 5,11 | ‘Loadel’ | 99,12-155 | 10 | US |
| CA Pop 5,16 | ‘O'Henry’ | F8,1-42 | 18 | US |
| CA Pop 5,17 | ‘Carson’ | *P. persica x P. davidiana* | 20 | US |
| CA Pop 5,18 | 2001,7-180 | 2001,7-180 | 17 | US |
| CA Pop 5,20 | 2000,3-205 | 2000,3-205 | 2 | US |
| CA Pop 7,12 | 2000,16-133 | 2000,16-133 | 14 | US |
| CA Pop 7,13 | 2000,3-205 | 2000,3-205 | 4 | US |
| CA Pop 8,13 | ‘Loadel’ | 2003,1-329 | 10 | US |
