# Supplementary material for: Development and Evaluation of a 9K SNP Array for Peach by Internationally Coordinated SNP Detection and Validation in Breeding Germplasm
Source: PLoS One. 2012 Apr 20;7(4):e35668. doi: 10.1371/journal.pone.0035668 (PMC3334984; doi:10.1371/journal.pone.0035668)
Supplement: Table S3 — Performance of the IPSC peach 9 K SNP array v1 on (a) EU (n = 232) and (b) US (n = 477) evaluation panels. (DOCX) [file pone.0035668.s005.docx]

**Table S3**: Performance of the IPSC peach 9K SNP array v1 on (a) EU (n=232) and (b) US (n=477) evaluation panels.

(a)

| Chromo-some | No. of SNPs attempted | No. of polymorphic SNPs | | | | | | | No. of polymorphic RosCOS SNPs | No. of mono-morphic SNPs | No. of failed SNPs |
| --- | --- | --- | --- | --- | --- | --- | --- | --- | --- | --- | --- |
|  |  | Total | MAF <0.05 | MAF 0.05-0.1 | MAF 0.1-0.2 | MAF 0.2-0.3 | MAF 0.3-0.4 | MAF 0.4-0.5 |  |  |  |
| 1 | 1114 | 845 | 30 | 35 | 138 | 283 | 250 | 109 | 1 | 219 | 50 |
| 2 | 1396 | 1100 | 32 | 51 | 204 | 389 | 240 | 184 | 2 | 223 | 73 |
| 3 | 811 | 631 | 17 | 16 | 95 | 202 | 181 | 120 | 0 | 138 | 42 |
| 4 | 1619 | 1315 | 43 | 38 | 152 | 476 | 436 | 170 | 2 | 227 | 77 |
| 5 | 546 | 433 | 14 | 26 | 63 | 58 | 89 | 183 | 1 | 105 | 8 |
| 6 | 933 | 742 | 14 | 21 | 197 | 269 | 141 | 100 | 1 | 143 | 48 |
| 7 | 793 | 622 | 20 | 22 | 111 | 168 | 175 | 126 | 1 | 122 | 49 |
| 8 | 913 | 691 | 38 | 62 | 197 | 241 | 101 | 52 | 0 | 175 | 47 |
| Total | 8125 | 6379 | 208 | 271 | 1157 | 2086 | 1613 | 1044 | 8 | 1352 | 394 |

(b)

| Chromo-some | No. of SNPs attempted | No. of polymorphic SNPs | | | | | | | No. of polymorphic RosCOS SNPs | No. of mono-morphic SNPs | No. of failed SNPs |
| --- | --- | --- | --- | --- | --- | --- | --- | --- | --- | --- | --- |
|  |  | Total | MAF <0.05 | MAF 0.05-0.1 | MAF 0.1-0.2 | MAF 0.2-0.3 | MAF 0.3-0.4 | MAF 0.4-0.5 |  |  |  |
| 1 | 1114 | 872 | 37 | 30 | 218 | 213 | 192 | 182 | 2 | 195 | 47 |
| 2 | 1396 | 1083 | 41 | 53 | 209 | 321 | 199 | 260 | 2 | 208 | 105 |
| 3 | 811 | 649 | 24 | 30 | 105 | 184 | 150 | 156 | 1 | 129 | 33 |
| 4 | 1619 | 1298 | 41 | 23 | 346 | 369 | 197 | 322 | 1 | 224 | 97 |
| 5 | 546 | 430 | 16 | 24 | 45 | 72 | 125 | 148 | 3 | 90 | 26 |
| 6 | 933 | 770 | 37 | 45 | 209 | 192 | 138 | 149 | 2 | 124 | 39 |
| 7 | 793 | 634 | 27 | 21 | 101 | 159 | 155 | 171 | 1 | 118 | 41 |
| 8 | 913 | 694 | 32 | 65 | 208 | 214 | 88 | 87 | 0 | 161 | 58 |
| Total | 8125 | 6430 | 255 | 291 | 1441 | 1724 | 1244 | 1475 | 12 | 1249 | 446 |
